# Lipoarabinomannan antigenic epitope differences in tuberculosis disease subtypes

**DOI:** 10.1038/s41598-020-70669-9

**Published:** 2020-08-18

**Authors:** Ruben Magni, Fatlum Rruga, Fahad M. Alsaab, Sara Sharif, Marissa Howard, Virginia Espina, Brianna Kim, Benjamin Lepene, Gwenyth Lee, Mohamad A. Alayouni, Hannah Steinberg, Robyn Araujo, Fatah Kashanchi, Fabio Riccardi, Sargento Morreira, Antonia Araujo, Fernando Poli, Devan Jaganath, Fred C. Semitala, William Worodria, Alfred Andama, Alok Choudhary, William J. Honnen, Emanuel F. Petricoin, Adithya Cattamanchi, Raffaella Colombatti, Jacobus H. de Waard, Richard Oberhelman, Abraham Pinter, Robert H. Gilman, Lance A. Liotta, Alessandra Luchini

**Affiliations:** 1 Center for Applied Proteomics and Molecular Medicine, George Mason University, Manassas, VA USA; 2 Dipartimento di Salute della Donna e del Bambino, Laboratorio di Oncoematologia, Università di Padova, Padova, Italy; 3 College of Applied Medical Sciences, King Saud bin Abdulaziz University for Health Sciences, Al Ahsa, Saudi Arabia; 4 Ceres Nanosciences, Inc., Manassas, VA USA; 5 Department of Global Community Health and Behavioral Sciences, Tulane University, New Orleans, LA USA; 6 Department of Epidemiology, University of Michigan, Ann Arbor, MI USA; 7 University of Illinois Chicago, Chicago, IL USA; 8 Queensland University of Technology, Brisbane, QLD 4000 Australia; 9 School of Systems Biology, George Mason University, Manassas, VA USA; 10 Aid Health and Development Onlus, Bissau, Guinea-Bissau; 11 University of Tor Vergata, Rome, Italy; 12 Departamento de Tuberculosis, Instituto de Biomedicina “Dr. Jacinto Convit”, Universidad Central de Venezuela, Caracas, Venezuela; 13 Division of Pediatric Infectious Diseases, University of California, San Francisco, San Francisco, CA USA; 14 Division of Pulmonary and Critical Care Medicine, University of California, San Francisco, San Francisco, CA USA; 15 Department of Internal Medicine, Makerere University College of Health Sciences, Kampala, Uganda; 16 Infectious Diseases Research Collaboration, Kampala, Uganda; 17 Mulago National Referral Hospital, Kampala, Uganda; 18 Public Health Research Institute, New Jersey Medical School, Rutgers, The State University of New Jersey, Newark, NJ USA; 19 Department of Women’s and Child’s Health, Azienda Ospedaliera-Università di Padova, Padova, Italy; 20 One Health Research Group, Facultad de Ciencias de la Salud, Universidad de las Américas, Quito, Ecuador; 21 Laboratorio de Investigación en Enfermedades Infecciosas, Laboratorio de Investigación y Desarrollo, Facultad de Ciencias y Filosofía, Universidad Peruana Cayetano Heredia, Lima, Peru; 22 Asociación Benéfica PRISMA, Lima, Peru; 23 Program in Global Disease Epidemiology and Control, Department of International Health, Bloomberg School of Public Health, Johns Hopkins University, Baltimore, MD USA

**Keywords:** Paediatric research, Translational research, Diagnosis

## Abstract

An accurate urine test for diverse populations with active tuberculosis could be transformative for preventing TB deaths. Urinary liporabinomannan (LAM) testing has been previously restricted to HIV co-infected TB patients. In this study we evaluate urinary LAM in HIV negative, pediatric and adult, pulmonary and extrapulmonary tuberculosis patients. We measured 430 microbiologically confirmed pretreatment tuberculosis patients and controls from Peru, Guinea Bissau, Venezuela, Uganda and the United States using three monoclonal antibodies, MoAb1, CS35, and A194, which recognize distinct LAM epitopes, a one-sided immunoassay, and blinded cohorts. We evaluated sources of assay variability and comorbidities (HIV and diabetes). All antibodies successfully discriminated TB positive from TB negative patients. ROAUC from the average of three antibodies’ responses was 0.90; 95% CI 0.87–0.93, 90% sensitivity, 73.5% specificity (80 pg/mL). MoAb1, recognizing the 5-methylthio-d-xylofuranose(MTX)-mannose(Man) cap epitope, performed the best, was less influenced by glycosuria and identified culture positive pediatric (N = 19) and extrapulmonary (N = 24) patients with high accuracy (ROAUC 0.87, 95% CI 0.77–0.98, 0.90 sensitivity 0.80 specificity at 80 pg/mL; ROAUC = 0.96, 95% CI 0.92–0.99, 96% sensitivity, 80% specificity at 82 pg/mL, respectively). The MoAb1 antibody, recognizing the MTX-Man cap epitope, is a novel analyte for active TB detection in pediatric and extrapulmonary disease.

## Introduction

Tuberculosis (TB) accounts for more than 1.7 million deaths per year^1^. While most deaths could be prevented by early diagnosis and initiation of treatment, conventional diagnostic methods requiring sputum (Xpert Ultra real-time PCR, sputum microscopy) need specialized training and costly equipment, and are currently inaccessible to many underserved populations in countries where TB is endemic^1–3^. Non-invasive urine immunoassay screening tests for mycobacterial antigens are an attractive alternative to sputum testing, because they can be deployed in low resource settings^4^. Measurement of the urinary TB carbohydrate antigen lipoarabinomannan has proved valuable for HIV positive patients with active pulmonary TB who have low CD4 counts^5^.

Nonetheless the vast majority of patients (85%) with active pulmonary TB are HIV negative. In a previous study we used a nanotechnology-enhanced assay to document LAM in the urine of HIV negative TB positive patients; LAM concentration ranged between 5 and 5,000 picograms/mL^6^ and positively correlated with disease severity. While LAM detection in HIV negative TB patients was initially controversial, several groups have now proven that LAM can be detected if the immunoassay has sufficient sensitivity.

In order to promote LAM urinary tests for widespread population screening for active TB, a number of important challenges need to be addressed. Firstly, LAM is a complex molecule and differences in its antigenic properties in the body fluids of infected patients, and animal models, compared to LAM produced by mycobacteria grown in vitro have been observed^7^. It is not clear which of its epitopes would be suitable for a highly sensitive assay for both HIV positive and HIV negative patients across geographic regions that differ in the prevalence of genetic strains of mycobacterium. We compared three different high quality and well-characterized antibodies^7^, recognizing known LAM epitopes. Our cohort included TB positive patients, healthy and diseased controls, across five different countries: Peru, Guinea Bissau, Uganda, Venezuela, and the United States.

A second unresolved challenge is determining the absolute concentration range of LAM in urine for HIV negative and HIV positive active TB. This is mandatory to establish the required sensitivity and quantitative range for ongoing and future development of point of care testing methods^5,8^. In the present study we calibrated the immunoassay to deterimine accurate concentration levels of LAM in microbiologically confirmed positive patients, for three different categories of anti-LAM antibodies.

A third issue is the interference, or influence, of comorbidities other than HIV to alter the urinary LAM concentration. Quality control (QC) of urine has not been emphasized in past studies. Specific gravity, urinary tract infections, ketonuria, hematuria and glycosuria can all influence a urine test^9^. For our study set of 430 patients we performed a full urine QC analysis and evaluated the percent of urine samples that have abnormal urinalysis values. Of particular interest was glycosuria, because diabetes is growing in prevalence among TB patients.

A fourth unresolved question we addressed is the utility of urinary LAM testing in pediatric TB and extrapulmonary TB. The utility of urinary LAM has been previoulsy validated in EPTB patient living with HIV^5^; here we investigate the urinary LAM concentration range in EPTB patients who are HIV negative. The rate of progression and the mortality rate for children and adults with extrapulmonary disseminated disease are higher than patients with isolated pulmonary TB^10^. These populations cannot produce sputum for conventional diagnostic modalities, thus an accurate urine screening test for active TB in these patients is critically needed^2–4,10–12^. Previous studies of urinary LAM diagnostics conducted on pediatric TB patients yielded low sensitivity, but nevertheless showed the promise of extending urinary LAM testing from adults to children^13,14^. In this study, we investigated new LAM epitopes and we identified one epitope that translates well to children.

## Results

**Figure 1:**
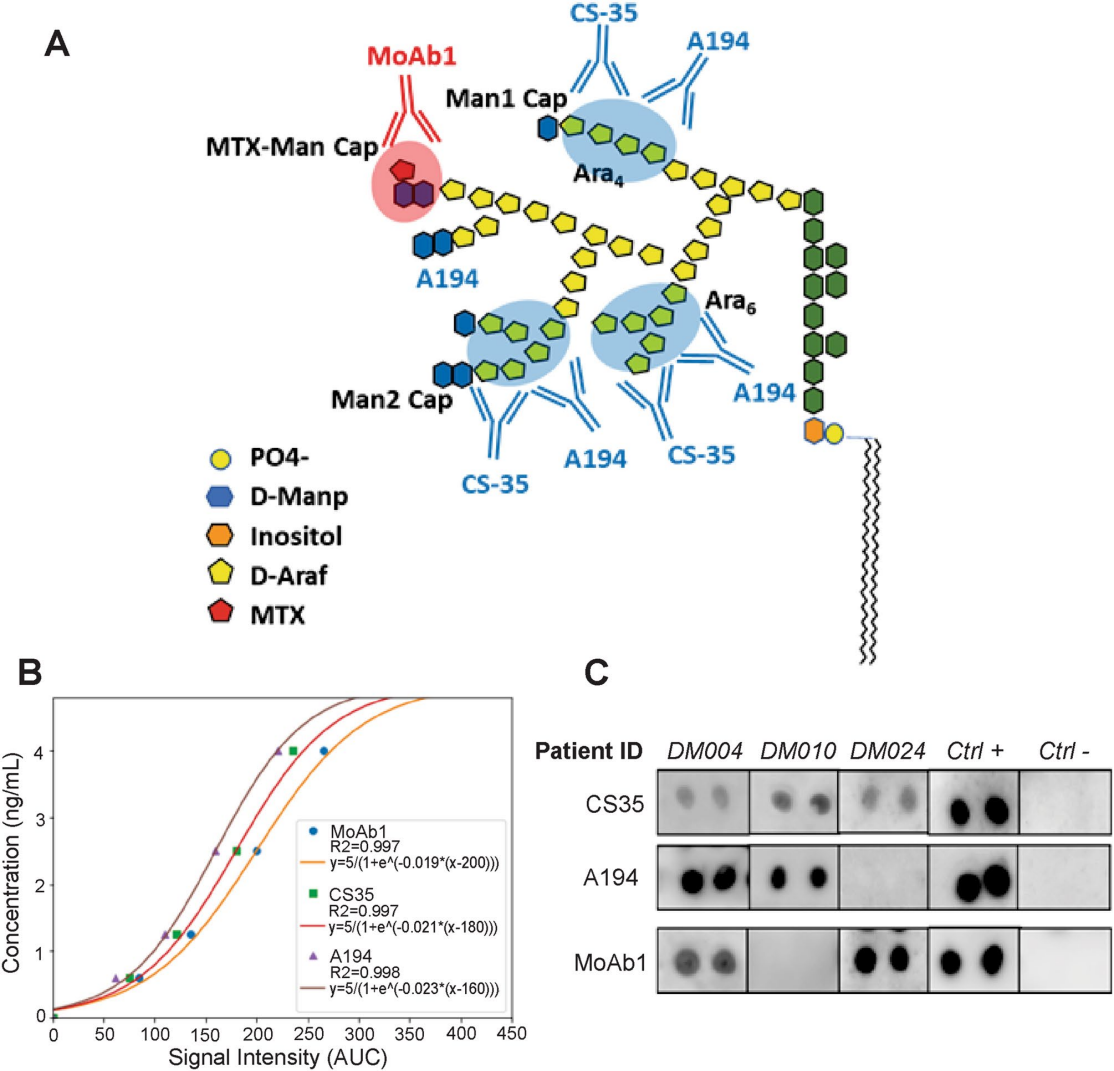
Characteristics of LAM immunoassays. (**A**) Monoclonal antibodies CS-35, A194 and MoAb1 recognize different LAM epitopes. Structural epitope configurations adapted from Sigal et al.^8^, Amin et al.^17^, and De et al.^18^. (**B**) Calibration curve of anti-LAM antibodies. Analytical measurement range for CS35, MoAb1, and A194 was 0.6–4 ng/mL in urine samples without dilution or concentration. (**C**) Example images show different mAb reactivity to the same urine sample cropped from different blots (Supplementary Figure S1).

Three anti-LAM antibodies MoAb1 IgG, CS35 IgG, A194 IgG were selected from a panel of 12 (Supplementary Table S1, Supplementary Methods) based on sensitivity, specificity and diversity of antigenic LAM epitopes using a previously published quantitative immunoassay described in Paris et al.^6^ (Fig. 1, Supplementary Fig. S1). Analytical measurement range in non-concentrated undiluted urine was 0.6–5 ng/mL. Nanocage pre-analytical concentration increased the sensitivity 50-fold. Limit of detection was 12 pg/mL pg/mL for MoAb1 and CS35, and 13 pg/mL for A194; limit of quantification was 43 pg/mL for MoAb1 and CS35, and 46 pg/mL for A194.

LAM concentration was measured with MoAb1, CS35 and A194 antibodies in a total of N = 219 TB patients and N = 211 controls. The median age of TB positive patients was 30 (IQR = 15) and 63% were male. Demographic, clinical and microbiological information are presented in Tables 1 and 2. The controls included healthy, PPD negative participants and symptomatic, PPD negative patients who were under evaluation for bacterial vector borne diseases (Table 1). Complete urinalysis was performed (Supplementary Table S2) and abnormal values were observed for glucose (24/430), bilirubin (47/430), ketone (26/430), blood (73/430), protein (158/430), urobilinogen (8/430), nitrites (6/430), and leukocyte esterase (78/430). Data results of the immunoassay analysis are reported in Supplementary Table S3.

**Table 1:** Demographic characteristics of sample cohort.

	No.	Median age, years (IQR)	Sex, ratio (M/F)
TB patients (microbiologically proven)	219	30 (15.25)	1.75
Healthy volunteers	106	28 (10.2)	1.2
Diseased TB negative controls	105	35.5 (14.25)	1.5

**Table 2:** Stratification of TB positive patients based on age, HIV status, glycosuria, drug-resistance, sputum smear grading, clinical manifestations, and geographic region.

TB positive patients	No.
Adults	200
Pediatric	19
**HIV**	
Positive	55
Negative	164
**Glycosuria**	
positive	24
negative	195
**Geographic area**	
Peru	80
Guinea Bissau	17
Uganda	97
Venezuela	25
Pleural biopsy acid fast bacilli positive	19
Ziehl-Neelsen sputum smear microscopy positive	11
**Auramine sputum smear microscopy**	
0	13
1	22
2	16
3	5
Paucibacillary	9
**Mycobacterium tuberculosis isolate resistant to**	
Isoniazid (only)	2
Multi-drug resistance	8
Pulmonary TB	195
**Extra-pulmonary TB **	24
Pleural	19
Laryngeal	1
Meningoencephalitis	1
Bone	1
Ganglionic	1
**PPD negative controls**	
Symptomatic (joint pain, fever, neurologic impairment, neuropathy, fatigue)	106
Healthy non-symptomatic	105

**Figure 2:**
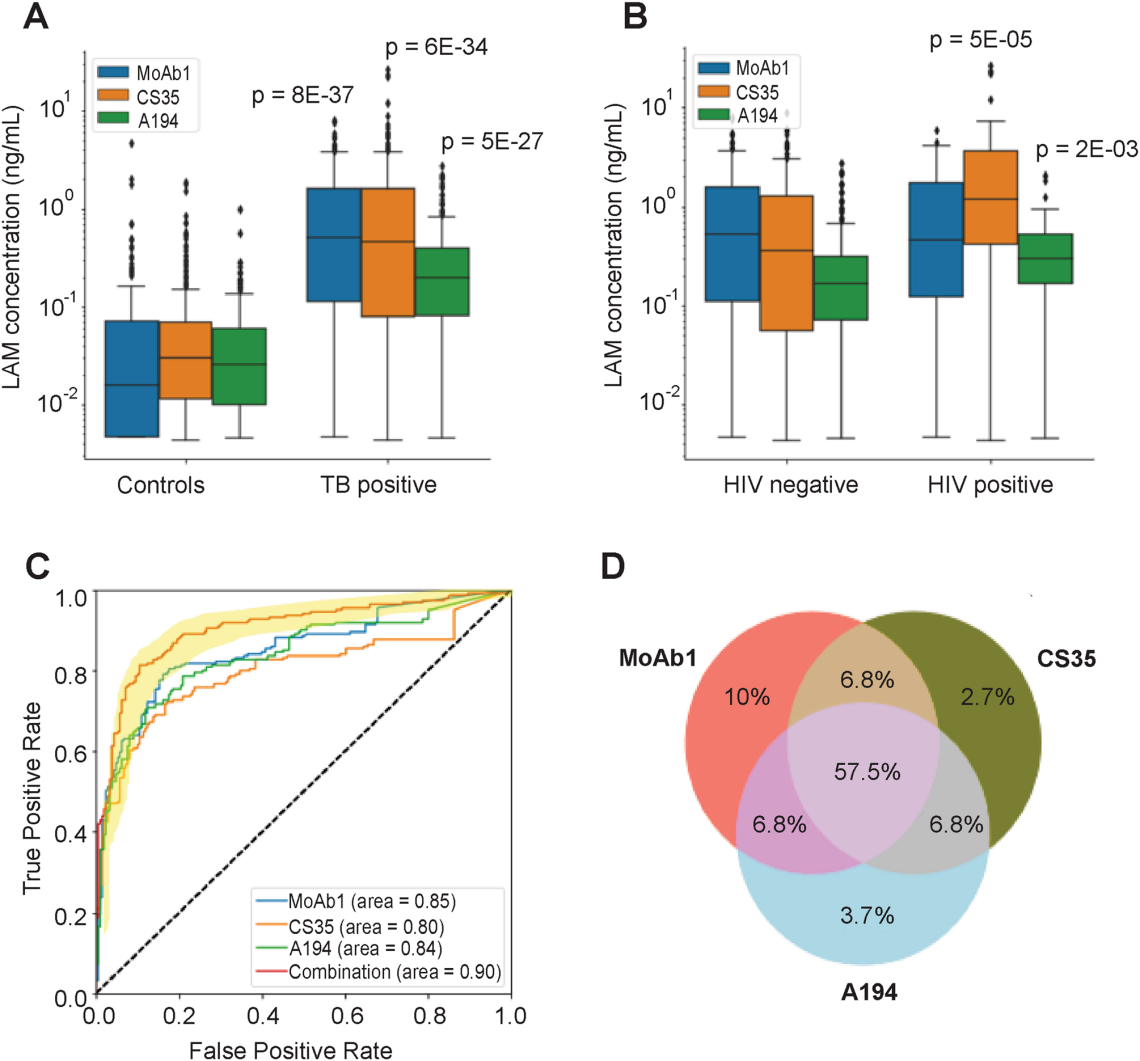
Urinary LAM discriminates TB positive from TB negative patients with high accuracy. (**A**) In a geographically diverse cohort (N = 430), urinary LAM concentration discriminates between cases and controls (Kruskal Wallis p = 8E−37, p = 5E−27, p = 6E−34, for MoAb1, CS35, A194 respectively). (**B**) Urinary LAM levels are higher in HIV+/TB+ patients than in HIV−/TB+ patients (Kruskal Wallis p = 5E−05, p = 0.002 for CS35 and A194, respectively). (**C**) Urinary LAM distinguishes microbiologically confirmed untreated TB positives from TB negatives with 90% sensitivity and 70% specificity, at a threshold of 80 pg/mL. (**D**) Urinary LAM antigenicity varies across samples. 57% of patients yield a signal for all three antibodies, whereas 95% of patients yield a signal to at least one antibody (cut-off 0.080 ng/mL).

The difference in LAM concentration between TB positive cases and TB negative controls was statistically significant (Kluskal–Wallis H test p = 8E−37, 6E−34, and 5E−27, for MoAb1, CS35, A194 respectively, Fig. 2) for all 3 antibodies. Median urinary LAM concentration in TB positive patients was 0.50 ng/mL (IQR = 1.50), 0.46 ng/mL (IQR = 1.52) and 0.20 ng/mL (IQR = 0.32) for MoAb1, CS35 and A194, respectively (Table 3). LAM concentration normalized by total protein concentration in the urine was 2.24 pg LAM/μg total protein (IQR = 6.67) for MoAb1, 2.40 pg LAM/μg total protein (IQR = 6.90) for CS35, and 0.9281 pg LAM/μg total protein (IQR = 2.10) for A194. Median urinary LAM concentration in TB negative patients was 0.016 ng/mL (IQR = 0.07), 0.03 ng/mL (IQR = 0.06), and 0.03 ng/mL (IQR = 0.05) for MoAb1, CS35 and A194, respectively.

**Table 3:** Concentration range of urinary LAM measured using MoAb1, CS35 and A194 mAbs, ng/mL (IQR).

Median LAM concentration, ng/mL (IQR)					
	Tot #	MoAb1	CS35	A194	Combination
TB positive	219	0.5 (1.50)	0.46 (1.52)	0.20 (0.32)	0.48 (1.08)
Adults	200	0.51 (1.60)	0.48 (1.57)	0.19 (0.31)	0.52 (1.10)
Pediatric	19	0.46 (0.91)	0.23 (0.62)	0.07 (0.41)	0.14 (0.24)
PTB	195	0.46 (0.54)	0.45 (1.54)	0.20 (0.31)	0.45 (1.00)
EPTP	24	1.40 (1.83)	0.84 (1.24)	0.194 (0.25)	0.73 (1.47)
**HIV status**					
Positive	55	0.46 (1.62)	1.19 (3.21)	0.30 (0.36)	0.79 (1.45)
Negative	164	0.52 (1.56)	0.55 (1.21)	0.13 (0.14)	0.40 (0.83)
**Glycosuria**					
Positive	24	0.76 (1.78)	0.21 (0.43)	0.11 (0.26)	0.44 (0.68)
Negative	195	0.49 (1.55)	0.56 (1.61)	0.20 (0.31)	0.47 (1.09)
**Countries**					
Peru	80	0.35 (1.19)	0.23 (0.89)	0.13 (0.22)	0.35 (0.66)
Guinea Bissau	17	0.54 (1.63)	0.004 (0.23)	0.031 (0.06)	0.31 (0.62)
Uganda	97	0.46 (1.30)	0.83 (2.22)	0.28 (0.37)	0.66 (1.39)
Venezuela	25	1.59 (1.78)	0.93 (1.17)	0.24 (0.40)	0.87 (1.46)

*PTB* pulmonary tuberculosis, *EPTB* extrapulmonary tuberculosis.

Sensitivity and specificity were evaluated by ROC analysis on all samples. MoAb1, CS35 and A194 yielded ROAUC of 0.85 (95% CI 0.82–0.89), 0.80 (95% CI 0.76–0.84), 0.84 (95% CI 0.80–0.88), respectively. By averaging the three antibodies values, ROAUC was 0.90 (95% CI 0.87–0.93), reaching 90% sensitivity and 73.5% specificity at a threshold of 80 pg/mL, which meets the requirements for a triage test according to WHO guidelines (Fig. 2). The cut-off threshold was identified using logistic regression and tenfold cross validation (Supplementary Fig. S2). In the cross validation, out-of-sample performance (AUC: 0.903, 95% CI 0.883–0.923) and classification error [0.186 (95% CI 0.156–0.216)] values were concordant with in-sample values (AUC: 0.903, 95% CI 0.900–0.906, *t* test *p* = 0.9807; error: 0.182, 95% CI: 0.175–0.189, *t-test* *p* = 0.8126) and showed that the models generalized well within the random, shuffled test-train splits.

We observed within-patient variability of antibody reactivity using titers normalized with individual dose response calibration to the BEI Resources LAM standard (Fig. 1c). 57.5% of TB patients were positive for all three antibodies using a cut-off of 80 pg/mL, and 5.5% of TB patients were negative for all three antibodies. Additionally, 6.8% of TB patients were positive for CS-35 and A194 but not for MoAb1, 6.8% were positive for MoAb1 and CS-35 but not for A194, and 6.8% were positive for MoAb1 and A194 but not for CS-35. 10%, 2.7%, and 3.7% of TB positive patients were positive solely for MoAb1, CS-35 and A194, respectively (Fig. 2).

LAM concentration was higher in HIV+/TB+ patients, compared to HIV−/TB+ patients. This well documented phenomenon may reflect a higher burden of *Mycobacterium* *tuberculosis* (*Mtb*) organisms in immunocompromised patients. Median LAM concentrations among TB+/HIV+ patients were 0.46 ng/mL (IQR = 1.62), 1.19 ng/mL (IQR = 3.21), 0.30 ng/mL (IQR = 0.36) for MoAb1, CS35, A194 respectively. Median LAM concentrations in TB+/HIV− patients were 0.52 ng/mL (IQR = 1.56), 0.35 ng/mL (IQR = 1.21), 0.16 ng/mL (IQR = 0.14) for the 3 antibodies respectively (Fig. 2, Table 3). The difference in LAM concentration between HIV+/TB+ patients, and HIV−/TB+ patients was statistically significant (p = 5E−05, p = 2E−03) for CS35 and A194 respectively but not for MoAb1 (Fig. 2). The LAM concentration range spanned over 4 orders of magnitude from values below the limit of detection to greater than 10 ng/mL, with higher concentrations generally distributed among the HIV+ patients (Fig. 2).

**Figure 3:**
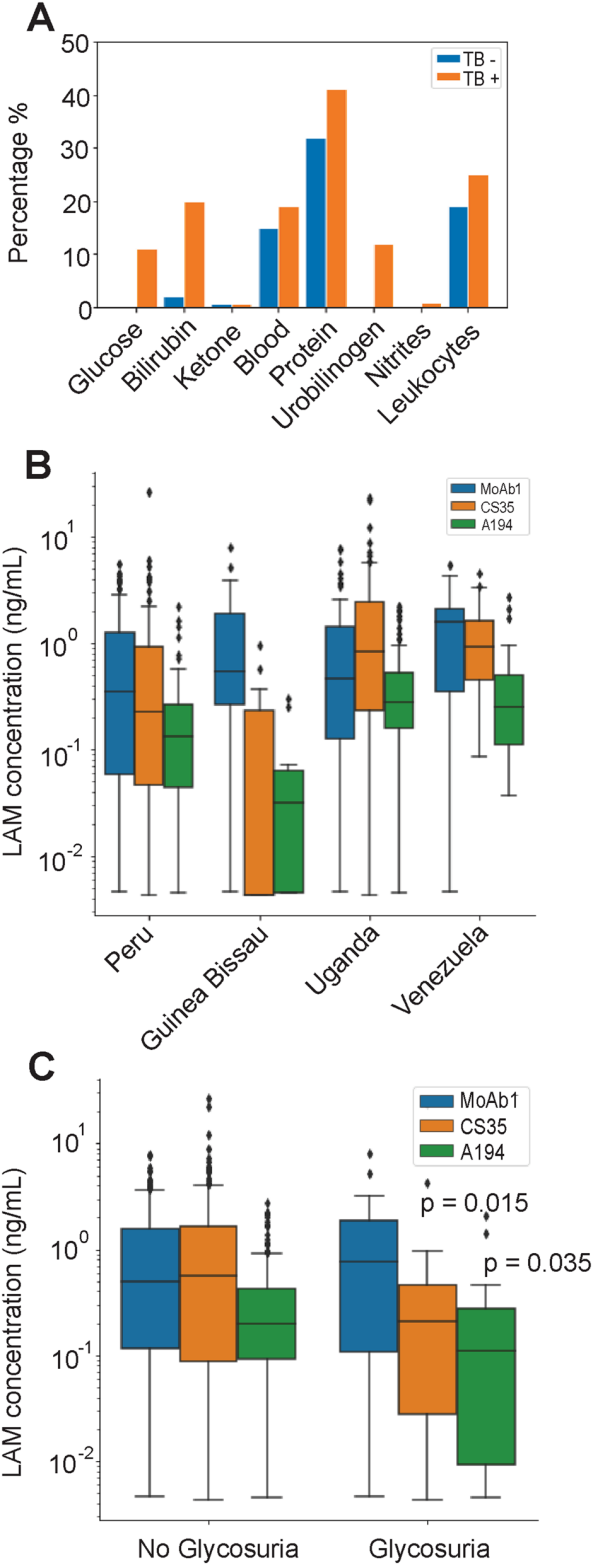
Urinalysis and geographical diversity influence LAM values. (**A**) Abnormal urine values were noted in both controls and TB positive patients. (**B**) Median urinary LAM concentration varies across different geographic areas (independent sample median test p = 0.030, pairwise comparison in Supplementary Figure S4). Assuming no collection and storage bias, this might be caused by different levels of disease severity at diagnosis, since access to healthcare might be delayed to a greater degree in certain countries. (**C**) Urinary LAM values were lower in TB patients with glycosuria (n = 22) when measured using CS-35 (p = 0.015) and A194 (p = 0.035).

Urinalysis was performed to understand the sources of variability for LAM testing. No sample was discarded on the basis of abnormal urinalysis values (e.g. glycosuria, bilirubinuria, hematuria, leukocytes, proteinuria) (Fig. 3). Among these variables, glycosuria was negatively associated with LAM concentration. LAM concentration was reduced in the presence of glycosuria using CS35 and A194 (p = 0.015, p = 0.035, respectively Fig. 3) while the effect of glycosuria was less prominent for MoAb1 (Fig. 3). LAM median concentration in patients with glycosuria was 0.76 ng/mL (IQR = 1.78), 0.21 ng/mL (IQR = 0.43), 0.11 ng/mL (IQR = 0.26) while in non-glycosuric TB positive patients was 0.49 ng/mL (IQR = 1.55), 0.56 ng/mL (IQR = 1.61), 0.20 ng/mL (IQR = 0.31) for MoAb1, CS35, A194, respectively (Table 3). A positive correlation was associated with abnormal proteinuria and LAM concentration for all three antibodies (logistic regression, MoAb1: Coef. odds ratio = 5.87, p = 9E−24, CI 1.47–2.08; CS35: odds ratio = 10.59, p = 2E−10, CI 1.67–3.06; A194: Coef. 1.63, p = 3E−19, CI 0.39–0.58) (Supplementary Table S4). MoAb1 retained high discriminatory power for TB positive patients with glycosuria versus negatives (AUC = 0.88, sensitivity = 87.5%, specificity = 76%, threshold = 80 pg/mL) (Supplementary Fig. S3).

Values of urinary LAM were analyzed in relation to country of origin, which included: Peru (N = 80), Guinea Bissau (N = 17), Uganda (N = 97), and Venezuela (N = 25). LAM concentration varied depending on geographic area (p = 0.029, p = 5E−6, p = 8E−6 for MoAb1, CS35, A194, respectively, Supplementary Fig. S4). Venezuela had the highest median concentration of 0.87 ng/mL (IQR = 1.56), followed by Uganda, 0.66 ng/mL (IQR = 1.39), Peru 0.35 ng/mL (IQR = 0.65) and Guinea Bissau 0.32 ng/mL (IQR = 0.62) (Fig. 3).

Since both glycosuria and geographical location quantitatively affected the assay (Fig. 3), we investigated their correlation. The frequency of glycosuria was higher (Chi square p = 2.058e−05) in patients from Guinea Bissau and Peru (35% and 19%), who are characterized by lower LAM values, than in Ugandan and Venezuelan patients (2% and 4%), who have higher LAM values. In an ANOVA model for multiple linear regression, the glycosuria:geographical location interaction term was significant for CS35 (p = 0.002), and for the average antibody signal (p = 0.004), but not for A194 (p = 0.14) and MoAb1 (p = 0.07). Therefore, glycosuria accounted for some of the geographical differences for the CS35 antibody, for the average antibody signal, but not for A194 and MoAb1. Thus antibody sugar epitope category was differentially sensitive to glycosuria.

**Figure 4:**
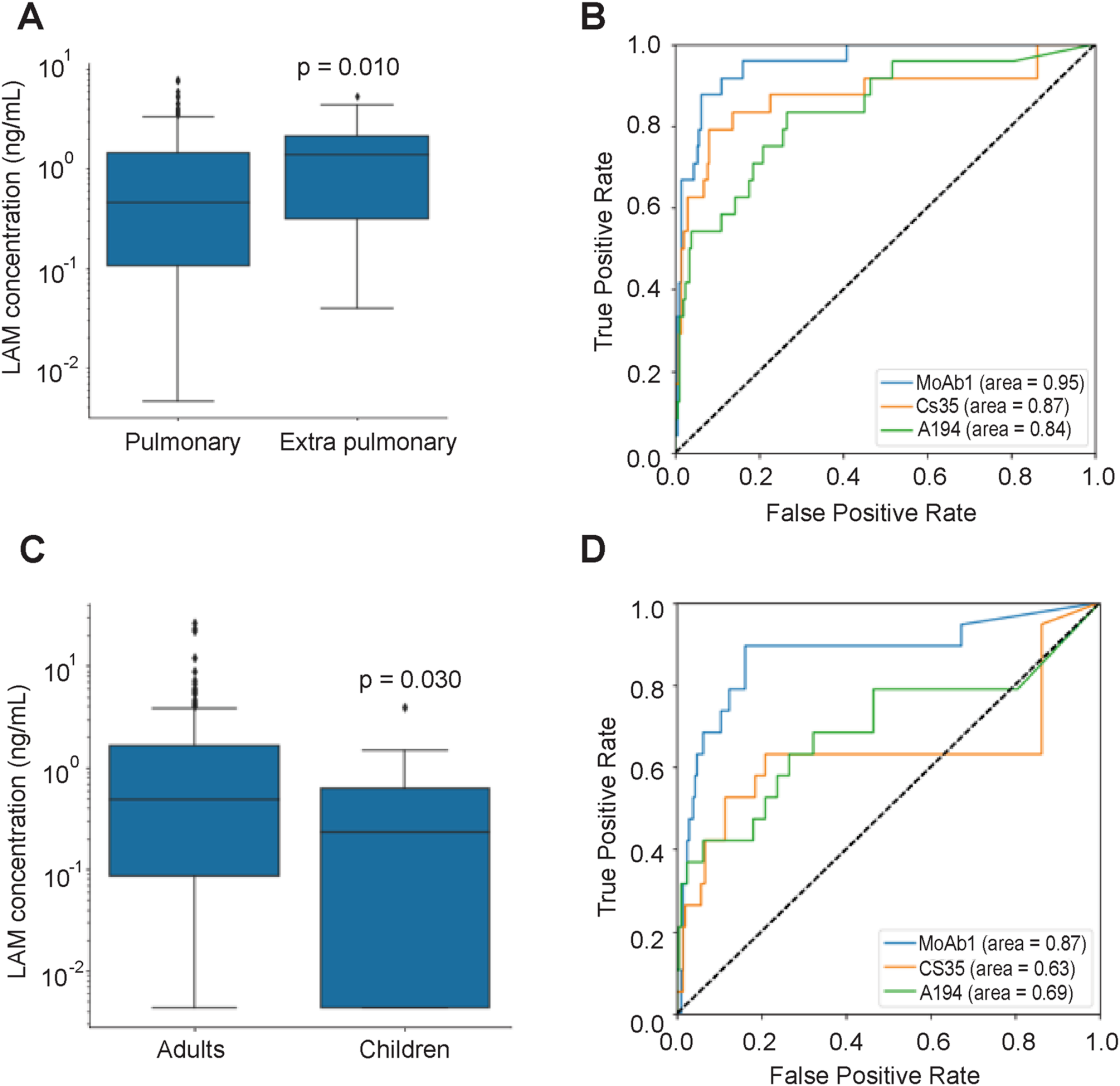
Urinary LAM is present at detectable concentrations in extrapulmonary adult TB patients and in microbiologically confirmed pediatric TB patients. (**A**) Levels of urinary LAM as measured using MoAb1 are significantly higher in extrapulmonary patients with respect to pulmonary patients. (**B**) MoAb1 is superior in discriminating extrapulmonary patients from non TB controls. (**C**) Levels of urinary LAM measured using CS35 are lower in TB positive pediatric patients than in TB positive adults. (**D**) ROC analysis demonstrated that MoAb1 was superior to the other antibodies in discriminating pediatric TB patients from negative controls.

The present dataset included two types of non-sputum producing patients: extrapulmonary disease (EPTB) and pediatric patients. Urinary LAM was detected with at least one antibody in all 19 pediatric TB patients analyzed (Supplementary Table S3). CS35 showed that LAM concentration in pediatric patients (0.23 ng/mL, IQR = 0.62) was significantly lower compared to adults (0.48 ng/mL, IQR = 1.57) (Fig. 4) (p = 0.030). MoAb1 discriminated well pediatric patients from negative controls (Fig. 4), with a ROC AUC of 0.87 (95% CI 0.77–0.98), and sensitivity and specificity of 0.90 and 0.80, respectively at a threshold of 80 pg/mL. For N = 24 EPTB cases, including pleural effusion, lymph node, bone, laryngeal, and meningoencephalitis TB, MoAb1-LAM signal was above the limit of detection for all samples, whereas CS-35 and A194 signals were negative in 2/24 and 1/24 EPTB patients, respectively. MoAb1 showed higher signal in EPTB (1.40, IQR = 1.83) compared to pulmonary TB (PTB) patients (0.46, IQR = 0.54) (p = 0.010) (Fig. 4). MoAb1 identified EPTB patients with high accuracy (ROAUC = 0.96, 95% CI 0.92–0.99, Fig. 4) reaching 96% sensitivity and 80% specificity at a threshold of 82 pg/mL.

## Discussion

We assessed whether a urinary LAM test for active TB could be extended beyond patients who are HIV coinfected. This study addressed several unresolved issues including the choice of antibodies, sources of assay variability, interfering comorbidities, and utility for childhood and extrapulmonary TB. We employed our previously described laboratory assay^6^ to assess the range of LAM urine in pre-treatment, microbiologically confirmed TB patients (N = 219), including HIV negative (N = 164) and HIV positive (N = 55) TB, pediatric (N = 19), extrapulmonary TB (N = 24), and controls (N = 211) including healthy (N = 105) and diseased (N = 106) from a range of geographical areas (United States, Venezuela, Guinea Bissau, Peru, Uganda).

It has recently been proposed that antigenically modified, or masked forms of the heterogeneous LAM molecule may be present in urine of infected patients. We cannot exclude that the LAM molecule might be altered depending on the stage of the disease, the host comorbidities, or the organ distribution of the infection. Since variability in the branched LAM structure has been previously described^7^ we addressed the performance of CS35, MoAb1 and A194, three different purified previously characterized^7^ anti-LAM mAbs, which recognize different sets of LAM epitopes^7^ (Fig. 1, Supplementary Methods) using our previously described quantitative dot blot, which concentrates and immobilizes all forms of urinary LAM onto a solid phase for probing with a single species of anti-LAM antibody.

Urinary LAM measurements successfully classified cases and controls with high significance (p = 8E−37, p = 5E−27, p = 6E−34, for MoAb1, CS35, A194, respectively) with a cut point of 80 pg/mL using CS35, MoAb1 and A194 for both HIV negative and HIV positive patients. Median LAM concentration values were 0.51 ng/mL, 0.46 ng/mL and 0.20 ng/mL for MoAb1, CS35, and A194 respectively. ROAUC was 0.85, 0.80, 0.84, respectively and ROAUC from the combination of the response from the three antibodies was 0.90, reaching 90% sensitivity at 73.5% specificity, confirming the utility of urinary LAM screening for active TB beyond patients with HIV co-infection.

Geographic location can introduce variability for TB screening because of heterogeneity in TB strain or clade prevalence, climate and state of hydration, disease stage at urine collection, comorbidity rate, and additional unknown factors. MoAb1 yielded higher median values than the other antibodies in Peru (N = 80), Guinea Bissau (N = 17) and Venezuela (N = 25). CS35, on the other hand, exhibited the highest median values in the Ugandan cohort (N = 97). MoAb1 showed the best discrimination performance in the Peru and Guinea Bissau sets. A194 yielded the best discriminatory power in Ugandan, and CS35 in Venezuelan patients. This inter-country difference was not entirely explained by difference in the frequency of HIV co-morbidity. For example, Venezuelan samples, which were all HIV negative, had the highest median value for CS35 and MoAb1 (Fig. 3). It is important to note that even if the median LAM concentration varied among countries, the discriminatory power for TB cases versus controls was maintained (Peru: ROAUC = 0.88; Guinea Bissau: ROAUC = 0.89; Uganda: ROAUC = 0.90; Venezuela: ROAUC = 0.90). This is an encouraging finding supporting expanded studies for worldwide screening.

Urinary sample quality and concentration state can have a profound effect on a screening assay. In the present study, approximately 20% of cases and controls had abnormal urinalysis values. The most significant endogenous interfering substance was glucose. The prevalence of type 2 diabetes mellitus (DM) is steeply increasing worldwide, in particular in low income countries where TB is endemic and the effect of glycosuria and diabetes on urinary LAM have not previously studied. Urinary LAM concentration in TB positive patients with glycosuria (n = 18) was significantly lower than TB positive patients with normal urinary glucose levels for CS35 (p = 0.015) and A194 (p = 0.035), but not for MoAb1 (Fig. 3). This finding is in keeping with reports suggesting that the *Mtb* cell wall polysaccharide composition is altered in diabetic patients^19^. MoAb1, which recognizes the MTX-Man cap of LAM, successfully discriminated TB positive patients presenting with glycosuria from TB negative controls (AUC = 0.87).

As previously observed^20–24^, LAM concentration was higher in HIV+/TB+ patients (CS35 p = 5E−05, A194 p = 0.0021). Patients with HIV co-infection were collected in Uganda (N = 36), Peru (N = 13), and Guinea Bissau (N = 6). 4/55 HIV co-infected patients were children. These covariates can increase the heterogeneity of LAM concentration values. In our previous study by Paris et al.^6^, we demonstrated that it is possible to detect urinary LAM in HIV negative TB patients using an experimental workflow which included a pre-analytical concentration step. Other investigators confirmed these findings^7,8,17,18,25^. Recent reports of LAM immunoassays employing high affinity purified antibodies indicate that acceptable analytical sensitivity required for HIV negative TB positive patients can be achieved without the use of a pre-analytical concentration step. The present study combined with the body of published work^6,8,26^ demonstrates that at least one of the tested antibodies has a sensitivity sufficient for a future field deployable test for HIV−/TB+ patients.

EPTB accounts for 15% of diagnosed TB cases^18^ and in its disseminated forms, leads to higher mortality rates compared to pulmonary TB^18^. In face of non-specific clinical symptoms and imaging tests, a definitive diagnosis of EPTB requires invasive and often impractical surgical procedures. We analyzed N = 24 EPTB HIV negative cases, which included pleural effusion, lymph node, bone, laryngeal, and meningoencephalitis TB. Three out of 24 patients were children. MoAb1 distinguished EPTB cases versus controls with high accuracy (AUC = 0.96, sensitivity = 96%, specificity = 80%, cut-off = 82 pg/mL, Fig. 4). A higher LAM signal can be explained by higher burden of disease that results in lower treatment success rates, which generally characterizes EPTB and disseminated TB patients^4^. In a previous study^6^, we have demonstrated correlation between urinary LAM values and severity of TB symptoms. Thus, the MoAb1 based LAM assay is a promising new approach to non-invasive detection of extrapulmonary disease.

Tuberculosis is among the top 10 causes of childhood mortality worldwide with a global estimate of 130,000 pediatric deaths per year^27^. Lack of appropriate screening, diagnostic, and treatment protocols has a severe toll on the life of TB pediatric patients: it is estimated that 1 in 4 undiagnosed children will die of the disease^19^. Because of the hurdle to procure invasive samples such as induced sputum and gastric lavage to perform *Mtb* culture, the need to develop sensitive technologies that enable *Mtb* detection non-invasively is of high priority to address the pediatric TB epidemic. We analyzed 19 culture positive pediatric patients (age, years: 2–17). 17/19 were positive for MoAb1, 12/19 and 9/19 were positive for A194 and CS35, respectively using a cut off of 80 pg/mL. MoAb1 showed great promise for pediatric screening (AUC = 0.87, sensitivity = 90%, specificity = 80%, cut-off = 80 pg/mL).

The present study has several weaknesses. Our goal was to mimic real-world urine testing conditions. Consequently, all samples, including those with abnormal urinalysis (e.g. glycosuria, bilirubinuria, hematuria, leukocytes, proteinuria) were included. In our patient cohort TB negatives are either asymptomatic, or symptomatic for non-TB diseases, but are not matched quantitatively in each country. A further limitation is that we used a one-sided immunoassay as opposed to a sandwich assay. Thus, while it can achieve a calibrated accurate concentration value for LAM, each antibody has to be judged against its own dose response curve. The overall combined sensitivity and specificity we observed for these heterogeneous cases for all countries combined was somewhat lower than we observed in our previous study of only HIV negative, TB culture positive, pre-treatment patients, from one hospital in one country^28^. Higher sensitivity and specificity were achieved when we subdivided the data by country of origin (Supplementary Figure S5). These regional comparisons imply potential variability in stage of disease or time of day for urine collection. Overall, across the heterogeneity of geographic regions, sample collecting institutions, urinary LAM continued to show strong promise for screening for patients with active TB, for assays that are adequately sensitive (LOD 12 pg/mL).

This study demonstrates that LAM as recognized by MTX-Man cap specific antibodies is a previously unappreciated, promising marker for pediatric and extrapulmonary disease patients. These findings support assay validation in larger cohorts of extrapulmonary and pediatric patients.

## Methods

### Study design and participants

In this case–control study, we assessed urine samples collected from adults and children undergoing evaluation for pulmonary or extra-pulmonary tuberculosis in four countries (Uganda, Peru, Venezuela, and Guinea Bissau). The George Mason University Institutional Review Board approved the study described herein (IRBnet IDs 1317042, 1244866, and 869592). The study followed principles of the Declaration of Helsinki and guidelines reported in Supplementary Methods. We included as cases patients with a confirmed diagnosis of pulmonary or extra-pulmonary TB based on positive mycobacterial culture results, smear microscopy, GenXpert, or biopsy. Adult patients provided written informed consent; minors (including children below 16 years) were enrolled in the study after obtaining written informed consent from parental/guardian or Legal Authorized Representative. Details about patient eligibility in each country are reported in Supplementary Methods. The cases included (1) N = 179 adults with pulmonary TB (N = 90 from Uganda, N = 78 from Peru, N = 4 from Venezuela and N = 7 from Guinea Bissau); (2) N = 19 pediatric patients, of whom 17had pulmonary TB (N = 7 from Guinea Bissau, N = 7 from Uganda, N = 2 from Venezuela) and 3 had extrapulmonary disease (N = X from Guinea Bissau); and (3) N = 24 patients with extra-pulmonary TB, of whom 21 were adults and 3 were pediatric (N = 2 from Peru, N = 3 from Guinea Bissau, N = 19 from Venezuela). The extra-pulmonary TB cases included N = 19 with pleural, N = 1 with meningeal, N = 1 laryngeal, N = 2 bone, and N = 1 ganglionic tuberculosis.

We included purified protein derivative (PPD) skin test-negative, healthy individuals from the United States, Uganda and Peru as non-symptomatic, TB negative controls. Diseased controls were PPD negative, symptomatic patients who were referred by infectious disease clinics in the United States, Peru and Uganda (Supplementary Methods).

### Procedures

Nanocage fabrication, covalent incorporation of binding baits, and one sided immunoassay was performed as described in Paris et al.^6^ and in the Supplementary Methods*.* Lipoarabinomannan preparation from *Mycobacterium* *tuberculosis* (*Mtb*) strain H37Rv (BEI Resources, catalog #NR-14848) was analyzed using the Anthrone method^15^ (Supplementary Fig. S6) and used as calibrator for the immunoassay.

Anti-LAM monoclonal antibodies CS35, A194, and MoAb1 were produced and purified according to Choudhary et al.^7^. CS-35 monoclonal antibody (mAb) was obtained from hybridoma cells derived from mice immunized with cell wall extracts of *M.* *leprae*. MoAb1 mAb was identified by phage display of single-chain variable fragment libraries generated from rabbits (Otsuka Pharmaceutical). A194 mAb was generated using in vitro cultures of fractionated memory B cells from a patient diagnosed with pulmonary TB. FIND 28 monoclonal antibody was provided by FIND^7^. All antibodies were purified using protein A chromatography and biotinylated.

LAM immunoassay was performed (Supplementary Methods) by spotting 2 µL of each sample in duplicate on polyvinylidene fluoride membranes (PVDF, Biorad) previously activated with methanol and equilibrated in DI water. Spots were allowed to dry and membranes were incubated with biotinylated anti-LAM MoAb1, CS-35, A194 mAbs and streptavidin-HRP (Biorad), and imaged using enhanced chemiluminescence system (Super- Signal West Dura, Thermo Fisher Scientific) and an Azure c300 imager. Densitometry analysis of immunoassay signals was conducted using ImageQuant software. Negative urine samples (N = 20) collected from healthy volunteers were considered as blanks and were used to calculate the background signal. The limit of detection (LOD) was calculated as the average of the blanks plus 2 standard deviations and the limit of quantification (LOQ) was the blank mean plus 10 standard deviations^16^.

### Statistical analysis

Patients were grouped by binary levels of categorical variables representing TB status, HIV status, pulmonary or extrapulmonary disease, and age (pediatric under NIH criteria of 18 years and younger versus adults). Statistical differences in urinary LAM values between the two groups were assessed by the Kluskal–Wallis H test with an alpha level of 0.05. Significance of LAM concentration differences among countries was assessed using the median test followed by a pairwise comparison by the Kluskal–Wallis H test. The analysis was performed using SPSS version 26. Relationship between covariates and outcome was investigated performing logistic and linear regression in Python 3 using the Scikit-Learn library. Covariates of interest included urinalysis measurements of glucose, bilirubin, ketone, blood, protein, urobilinogen, nitrite and leukocyte esterase, auramine staining scores, Microscopic Observation Drug Susceptibility Assay (MODS) results, MODS time to positivity. LAM concentration was considered as linear outcome. For the logistic regression, LAM measurements obtained with different antibodies were considered as covariates against TB status as binary outcome. Statistical significance of regression coefficients was assessed by two-sided t tests and z tests with an alpha level of 0.05. Receiver operating characteristics (ROC) analysis was performed using Python Scikit-Learn library; confidence intervals were calculated with 1,000 stratified bootstrap replicates. The urinary LAM concentration cut off was defined using logistic model regression, ROC analysis and tenfold cross validation studies (Supplementary Fig. S2). Visualization of box plot, ROC plots, and Venn Diagrams were obtained using Python 3 Matplotlib 3.1.1 and Seaborn 9.0 libraries.

## Supplementary information

Supplementary file1
