# Author Correction: Lipoarabinomannan antigenic epitope differences in tuberculosis disease subtypes

**DOI:** 10.1038/s41598-021-98304-1

**Published:** 2021-09-27

**Authors:** Ruben Magni, Fatlum Rruga, Fahad M. Alsaab, Sara Sharif, Marissa Howard, Virginia Espina, Brianna Kim, Benjamin Lepene, Gwenyth Lee, Mohamad A. Alayouni, Hannah Steinberg, Robyn Araujo, Fatah Kashanchi, Fabio Riccardi, Sargento Morreira, Antonia Araujo, Fernando Poli, Devan Jaganath, Fred C. Semitala, William Worodria, Alfred Andama, Alok Choudhary, William J. Honnen, Emanuel F. Petricoin, Adithya Cattamanchi, Raffaella Colombatti, Jacobus H. de Waard, Richard Oberhelman, Abraham Pinter, Robert H. Gilman, Lance A. Liotta, Alessandra Luchini

**Affiliations:** 1 Center for Applied Proteomics and Molecular Medicine, George Mason University, Manassas, VA USA; 2 Dipartimento Di Salute Della Donna e del Bambino, Laboratorio di Oncoematologia, Università di Padova, Padova, Italy; 3 College of Applied Medical Sciences, King Saud Bin Abdulaziz University for Health Sciences, Al Ahsa, Saudi Arabia; 4 Ceres Nanosciences, Inc, Manassas, VA USA; 5 Department of Global Community Health and Behavioral Sciences, Tulane University, New Orleans, LA USA; 6 Department of Epidemiology, University of Michigan, Ann Arbor, MI USA; 7 University of Illinois Chicago, Chicago, IL USA; 8 Queensland University of Technology, Brisbane, QLD 4000 Australia; 9 School of Systems Biology, George Mason University, Manassas, VA USA; 10 Aid Health and Development Onlus, Bissau, Guinea-Bissau; 11 University of Tor Vergata, Rome, Italy; 12 Departamento de Tuberculosis, Instituto de Biomedicina “Dr. Jacinto Convit”, Universidad Central de Venezuela, Caracas, Venezuela; 13 Division of Pediatric Infectious Diseases, University of California, San Francisco, San Francisco, CA USA; 14 Division of Pulmonary and Critical Care Medicine, University of California, San Francisco, San Francisco, CA USA; 15 Department of Internal Medicine, Makerere University College of Health Sciences, Kampala, Uganda; 16 Infectious Diseases Research Collaboration, Kampala, Uganda; 17 Mulago National Referral Hospital, Kampala, Uganda; 18 Public Health Research Institute, New Jersey Medical School, Rutgers, The State University of New Jersey, Newark, NJ USA; 19 Department of Women’s and Child’s Health, Azienda Ospedaliera-Università di Padova, Padova, Italy; 20 One Health Research Group, Facultad de Ciencias de la Salud, Universidad de Las Américas, Quito, Ecuador; 21 Laboratorio de Investigación en Enfermedades Infecciosas, Laboratorio de Investigación y Desarrollo, Facultad de Ciencias y Filosofía, Universidad Peruana Cayetano Heredia, Lima, Peru; 22 Asociación Benéfica PRISMA, Lima, Peru; 23 Program in Global Disease Epidemiology and Control, Department of International Health, Bloomberg School of Public Health, Johns Hopkins University, Baltimore, MD USA

Correction to: *Scientific Reports* 10.1038/s41598-020-70669-9, published online 18 August 2020

The original version of this Article contained an error in the spelling of the author Fahad M Alsaab which was incorrectly given as Fahad Alsaab.

Additionally, the Affiliations given for author Fahad M. Alsaab were incomplete.

The correct affiliations are listed below.

Center for Applied Proteomics and Molecular Medicine, George Mason University, Manassas, VA, USA.

College of Applied Medical Sciences, King Saud bin Abdulaziz University for Health Sciences, Al Ahsa, Saudi Arabia.

As a result of the changes, the Affiliations have been renumbered.

The original Article and accompanying Supplementary Information file have been corrected.

