# Supplementary material for: Lipoarabinomannan antigenic epitope differences in tuberculosis disease subtypes
Source: Sci Rep. 2020 Aug 18;10:13944. doi: 10.1038/s41598-020-70669-9 (PMC7434769; doi:10.1038/s41598-020-70669-9)

# Supplementary Information for:

## Title: **Lipoarabinomannan antigenic epitope differences in tuberculosis disease subtypes.**

Ruben Magni^1^, Fatlum Rruga^1,2^, Fahad M. Alsaab^1,3^, Sara Sharif^1^, Marissa Howard^1^, Virginia Espina^1^, Brianna Kim^4^, Benjamin Lepene^4^, Gwenyth Lee^5,6^, Mohamad A Alayouni^1^, Hannah Steinberg^7^, Robyn Araujo^8^, Fatah Kashanchi^9^, Fabio Riccardi^10,11^, Sargento Morreira^10^, Antonia Araujo^10^, Fernando Poli^12^, Devan Jaganath^13,14^, Fred C. Semitala^15,16^, William Worodria^15,17^, Alfred Andama^15,16^, Alok Choudhary^18^, William J Honnen^18^, Emanuel F Petricoin III^1^, Adithya Cattamanchi^14^, Raffaella Colombatti^19^, Jacobus H. de Waard^12,20^, Richard Oberhelman^5^, Abraham Pinter^18^, Robert H. Gilman^21,22,23^, Lance A. Liotta^1^, Alessandra Luchini^1,^*.

*to whom the correspondence should be addressed: aluchini@gmu.edu

**Affiliations**

^1^Center for Applied Proteomics and Molecular Medicine, George Mason University, Manassas, Virginia, USA.

^2^Dipartimento di Salute della Donna e del Bambino, Laboratorio di Oncoematologia, Università di Padova, Padova, Italy.

^3^College of Applied Medical Sciences, King Saud bin Abdulaziz University for Health Sciences, Al Ahsa, Saudi Arabia.

^4^Ceres Nanosciences, Inc., Manassas, Virginia, USA.

^5^Department of Global Community Health and Behavioral Sciences, Tulane University, New Orleans, Louisiana, USA.

^6^Department of Epidemiology, University of Michigan, Ann Arbor, Michigan, USA.

^7^University of Illinois Chicago, Chicago, Illinois, USA.

^8^Queensland University of Technology, Brisbane, QLD, 4000, Australia.

^9^School of Systems Biology, George Mason University, Manassas, Virginia, USA.

^10^Aid Health and Development Onlus, Bissau, Guinea Bissau.

^11^University of Tor Vergata, Rome, Italy.

^12^Departamento de Tuberculosis, Instituto de Biomedicina “Dr. Jacinto Convit”. Universidad Central de Venezuela. Caracas, Venezuela.

^13^Division of Pediatric Infectious Diseases, University of California, San Francisco, San Francisco, CA USA.

^14^Division of Pulmonary and Critical Care Medicine, University of California, San Francisco, San Francisco, CA USA.

^15^Department of Internal Medicine, Makerere University College of Health Sciences, Kampala, Uganda.

^16^Infectious Diseases Research Collaboration, Kampala, Uganda.

^17^Mulago National Referral Hospital, Kampala, Uganda.

^18^Public Health Research Institute, New Jersey Medical School, Rutgers, The State University of New Jersey, Newark, New Jersey, USA.

^19^Department of Women's and Child's Health, Azienda Ospedaliera-Università di Padova, Padova, Italy.

^20^One Health Research Group. Facultad de Ciencias de la Salud. Universidad de las Américas, Quito, Ecuador

^21^Laboratorio de Investigación en Enfermedades Infecciosas, Laboratorio de Investigación y Desarrollo, Facultad de Ciencias y Filosofía, Universidad Peruana Cayetano Heredia, Lima, Peru.

^22^Asociación Benéfica PRISMA, Lima, Peru.

^23^Program in Global Disease Epidemiology and Control, Department of International Health, Bloomberg School of Public Health, Johns Hopkins University, Baltimore, Maryland, USA.

*to whom the correspondence should be addressed: aluchini@gmu.edu

**Table of Content:**

1. Supplementary methods 2
   1. Study guidelines
   2. Nanocage synthesis, Sample processing with nanocages, LAM immunoassay
   3. Epitope specificity of MoAb1, CS35 and A194
2. Supplementary Figures 5
   1. Figure S1. Original, unprocessed images of the blots presented in Figure 1C.
   2. Figure S2. Logistic regression and 10-fold cross validation studies were performed in order to define a urinary LAM cut off threshold.
   3. Figure S3. MoAb1 retained high discriminatory power for TB positives versus negatives (AUC 0.88) in glycosuric patients.
   4. Figure S4. Pairwise comparison of LAM concentration in TB positive patients grouped by country of origin.
   5. Figure S5. Urinary LAM successfully distinguishes microbiologically confirmed untreated TB positives from TB negatives in each country cohort.
   6. Figure S6. Total saccharide concentration in the LAM reference standard (BEI Resources).
3. Supplementary Tables 10
   1. Table S1. List of antibodies tested in this study.
   2. Table S2. Results from urine dipstick analysis of sample cohort using Siemens Multistix 10SG Reagents Strips.
   3. Table S3. Values of urinary LAM concentration in TB patients and controls (N=430) measured using MoAb1, CS35, and A194 antibodies.
   4. Table S4. Linear regression analysis shows that LAM concentration positively correlates with (p-value 0.000) with urinary protein content.

# Supplementary Methods

**Study guidelines.**

In this study, we assessed urine samples collected from tuberculosis patients under medical examination in four countries: Uganda, Peru, Venezuela, and Guinea Bissau. All urine specimens were collected prior anti-tuberculosis treatment initiation.

1. One set of Ugandan patient urine samples was collected from hospitalized and outpatient HIV-positive and HIV-negative adults (age >18 years, N=90) at Kiruddu General Referral Hospital, Makindye Division, Kampala, Uganda^27^. All study procedures were reviewed and approved by the Makerere University School of Medicine Research Ethics Committee, and the George Mason University ethical committee. Conditions for considering a patient positive for tuberculosis were: positive sputum culture via Mycobacterium growth indicator tube 960 (MGIT) system (Becton-Dickinson Microbiology Systems, Sparks, MA) or solid (Lowenstein Jensen) and positive sputum GeneXpert MTB/RIF assay. Patients were excluded if (1) Xpert results were indeterminate; (2) patients were presently taking or had taken in the past 12 mopernths anti-TB treatment or agents with anti-TB activity (e.g. fluoroquinolones); or (3) patients refused or were unable to provide informed consent.
2. The second set of Ugandan patients comprised tuberculosis pediatric patients aged six months to 5 years who were recruited in the Mulago Hospital (Uganda’s National Referral Hospital), Nsambya Hospital, and the HIV Family Care program that is based at the Makerere University –Johns Hopkins University clinic (N=7). All study procedures were reviewed and approved by the Makerere University School of Medicine Research Ethics Committee, the John Hopkins University Ethics committee, and the George Mason University ethical committee. Following saline nebulization, sputum was collected by expectoration, where possible, or through suctioning techniques. Sputum was then analyzed using acid-fast bacilli (AFB) smear, Lowenstein-Jensen (LJ) culture, Mycobacteria Growth Indicator Tube (MGIT), Microscopic Observation Drug Susceptibility (MODS), and GeneXpert. Patients were considered positive for tuberculosis if one of the aforementioned assays was positive. A child was eligible to participate in the study if the following criteria were met: 1) contact with known sputum smear positive PTB case, 2) cough of 2 or more weeks, 3) fever >38˚ C for 14 days after other causes such as pneumonia and malaria have been ruled out, 4) Documented weight loss or failure to gain weight. Exclusion criteria were: 1) unconsciousness or any other life-threatening condition which require immediate initiation of therapy, 2) unwillingness or impossibility to attend study visits as scheduled, 3) Current anti-tuberculosis therapy, and 4) initiation of antiretroviral therapy within the preceding 3 months. Purified protein derivative (PPD) skin test-negative, healthy individuals were enrolled as controls.
3. The Peruvian samples included adult tuberculosis patients (N=80) recruited in 2 reference tertiary academic Peruvian Hospitals: Hospital Nacional Dos de Mayo, Lima, and Hospital Nacional Daniel Alcides Carrión, Callao, Peru^28^. Patients were included in the study if they were MODS or smear microscopy positive. Patients were not eligible to participate if 1) less than 18 years of age, 2) pregnant as defined by positive results on serum or urine beta-human chorionic gonadotropin, 3) started TB treatment, 4) unable or unwilling to provide informed consent. One patient was diagnosed with laryngeal tuberculosis by biopsy, and one patient was diagnosed with meningoencephalitis by cerebrospinal fluid Ziehl-Neelsen (ZN) staining for acid-fast bacilli. Purified protein derivative (PPD) skin test-negative, healthy individuals were enrolled as controls.
4. Venezuelan patients were enrolled in the Laboratorio de Tuberculosis, Instituto de Biomedicina, Caracas, Venezuela. The study was approved by the ethical committee of Instituto de Biomedicina in Caracas, Venezuela. Pleural tuberculosis adult patients (N=19) were considered positive if they had a positive pleural biopsy AFB staining. Pulmonary tuberculosis patients were considered positive if they had a positive sputum smear AFB staining. N=2 pediatric patients (8 and 10 years old) were subjected to gastric aspiration and resulted positive to culture.
5. Patient samples from Guinea Bissau were obtained from The Hospital Raoul Follereau (HRF), located in Bissau, Guinea Bissau. Patients suspected of early or late stage TB were admitted to the HRF after referral from regional hospitals or health centers across the entire country. All patients were subjected to a medical examination and chest x-ray. Diagnosis of TB was made according to the routinely used clinical protocol from the National Guidelines.[^20^](https://www.ncbi.nlm.nih.gov/pmc/articles/PMC5667531/#b20-mjhid-9-1-e2017059) In patients with clinical and/or radiologic findings consistent with pulmonary TB, sputum-smear microscopy using Ziehl-Neelsen’s stain was performed. If bacilli were identified in at least two samples, patients were considered TB positive. If lymph node involvement was suspected, lymph node aspirate was used for acid fast bacilli (AFB) stain and microscopy examination; if osteoarticular involvement was suspected, appropriate skeletal radiology guided biopsies were performed.
6. Diseased controls in the United States were PPD negative, symptomatic patients who were referred by infectious disease clinics. Urine samples were collected from consented (George Mason University IRB 869592) number patients who were suspected of having tick-borne diseases in different geographic regions at high risk for tick-borne diseases in the US (clinics: Hope McIntyre, MD, Maryland; Deborah Hoadley MD LLC, Massachusetts). Acceptance criteria for acute Lyme Borreliosis (LB) patients included the characteristic erythema migrans (EM) rash and positive two-tier LB serology according to CDC criteria. Non acute patients suspected of tick-borne illnesses included post treatment Lyme disease syndrome (PTLDS) patients; acceptance criteria included previous LB diagnosis and persistence of symptoms following antibiotic therapy for LB according to the Infectious Disease Society of America guidelines (doi: 10.1016/j.ijid.2013.01.008). An informed consent form was signed by all patients enrolled in this study and by their treating physicians. If the patient was a minor, written consent and assent from the subject, was obtained from a parent or legal guardian. All methods were performed in accordance with relevant guidelines and regulations. Clinical and demographic data included age, sex, previous tick-borne disease diagnosis, self-reported symptoms, and physician assessed symptoms.
7. PPD-negative healthy, asymptomatic participants donated urine at George Mason University after signing an informed consent (Geroge Mason University IRB 1244866).

**Nanocage synthesis**

Briefly Poly(NIPAm-co-BAC-co-AA) N-Isopropylacrylamide (NIPAm, 4.5 g, 39 mmol), bis(acryloyl)cystamine (BAC 236 mg, 0.9 mmol) and allylamine (AA, 338 μl, 4.5 mmol) were dissolved in 150 mL of water, and filtered using a 0.45 μm nitrocellulose membrane disk filter. The solution was purged with nitrogen for 30 minutes at room temperature under stirring and then heated to 50°C. N,N,N',N'-tetramethylethylenediamine (TEMED, Thermo Fisher, 19.4 mg, 0.17 mmol) was added to the solution and after 10 minutes potassium persulfate (50 mg, 0.18 mmol) was added to initiate the polymerization. The solution was held at 50°C under nitrogen for 4 hours prior to washing and size characterization by light scattering. Reactive blue 221 (RB221), trypan blue (TB) were covalently incorporated in the nanoparticles. RB221 was coupled as follows: 0.3 g of RB221 powder was mixed to a solution obtained by adding 0.66 g of Na_2_CO_3_ to 50 ml of DI water and stirring at medium rate until completely dissolved. The solution was filtered (0.45 μm pore size). 50 ml of cage suspension was added and allowed to incubate overnight at room temperature. RB221 coupled cages were washed five times (54,400 rcf, 50 min, 25 °C) and re-suspended in 50 mL of DI water. Trypan Blue was coupled as follows: 0.3 g of Trypan Blue powder was mixed with water pH4 and the solution is filtered. 50 ml of cage suspension was added and incubated overnight at 50ºC. Trypan blue coupled cages were washed five times (54,400 rcf, 50 min, 25 °C) and re-suspended in 50 mL of DI water.

**Sample processing with nanocages**

Urine samples were received in frozen state, thawed, and centrifuged for 5 minutes at 1000 rpm. Supernatants were recovered. In order to sterilize the sample, 50 ul of 10% sodium dodecyl sulfate (SDS) solution in water was added to 1 mL of patient urine and spiked-in controls. Samples were incubated for 5 minutes at 100ºC. Samples were then placed in ice for 20 minutes and centrifuged for 5 minutes at 5000 rcf. Supernatant was recovered and incubated with 100 µl of nanocage suspension (5 mg/ml, dry weight) for 30 minutes. Cages were separated from urine by centrifugation at 10,000 rcf for 10 min at 25°C and washed with de-ionized water. Cages were re-suspended with 15 µL of a solution of 2% SDS in water and incubated for 10 minutes at 100°C. Finally, cages were centrifuged at 10,000 rcf for 10min, and supernatant was recovered.

**LAM immunoassay**

Calibration curve was obtained spiking the LAM standard in pooled control urine from TB negative, healthy participants. LAM immunoblot was performed by spotting 2 µl of each sample in duplicate on polyvinylidene fluoride membranes (PVDF, Biorad) previously activated with methanol and equilibrated in DI water. Spots were allowed to dry and membranes were blocked with a solution of 0.2% I-Block (Applied Biosciences) and 0.1% Tween 20 (Fisher) in PBS (Life Technologies) for 45 minutes at room temperature under rotation. Membranes were incubated with biotinylated (Ab biotinylation was performed with Lightning-Link® Rapid Biotinylation Kit – Type A Cat#: 370-0010) anti-LAM MoAb1, CS-35, A194 diluted 1:2,000, 1:1,000 and 1:1,000, respectively in a solution of 0.2% I-Block and 0.1% Tween 20 in PBS overnight at 4 °C. Membranes were then washed in a solution of 5% non-fat dry milk and 0.1% Tween 20 in PBS for 30 minutes at room temperature under rotation, and incubated with streptavidin-HRP (Biorad) diluted 1:5,000 in a solution of 5% non-fat dry milk and 0.1% Tween 20 in PBS for 40 minutes at room temperature under rotation. Membranes were washed with a solution of 0.1% Tween 20 in PBS for 20 minutes at room temperature under agitation, and imaged using enhanced chemiluminescence system (Super- Signal West Dura, Thermo Fisher Scientific) and an Azure c300 imager. Densitometry analysis of immune-macroarray signals was conducted using ImageQuant software.

### **Epitope specificity of MoAb1, CS35 and A194**

*Mycobacterium tuberculosis* (*Mtb*) LAM comprises three regions: a glycosylphosphatidylinositol (GPI) anchor, a D-mannan backbone, and an immunologically active, highly branched, D-Arabinan domain. The arabinan domain contains two types of terminal chains: linear tetra-arabinoside (Ara-4) and branched hexa-arabinoside (Ara-6) structures. The non-reducing ends of the arabinan branches can be capped with multiple motif classes of monomannose, dimannose and trimannose (Man) with the possible further addition of a 5-methyl-thio-xylose (MTX) motif^11^

We selected 3 different antibodies, MoAb1 IgG, CS35 IgG, and A194 IgG that are known to recognize different regions of the LAM molecule. A194 and CS35 target Ara4/Ara6 structures which are shared by all mycobacterium species^11^, whereas MoAb1 targets MTX-Man caps, which were demonstrated to be specific for pathogenic mycobacteria including *Mycobaterium tuberculosis*^11^. MoAb1 is a humanized version of Otsuka antibody S4-20 described in Sigal er al (ref 12).

# SUPPLEMENTARY FIGURES


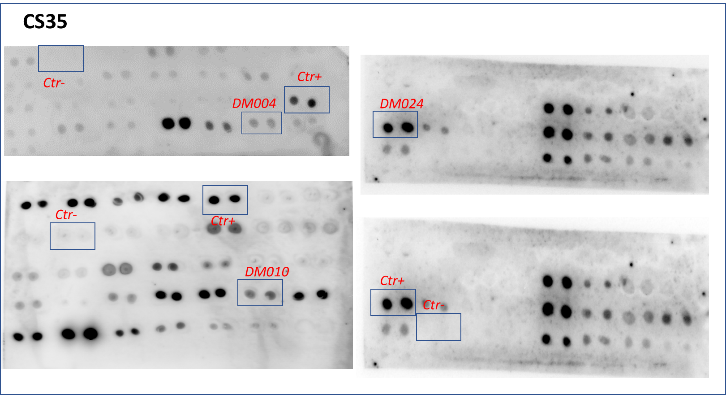

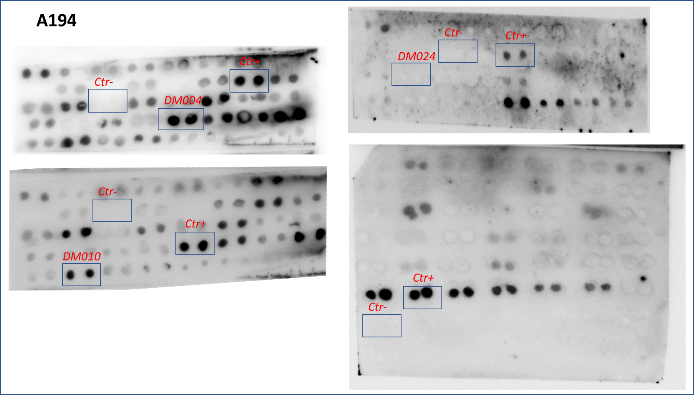


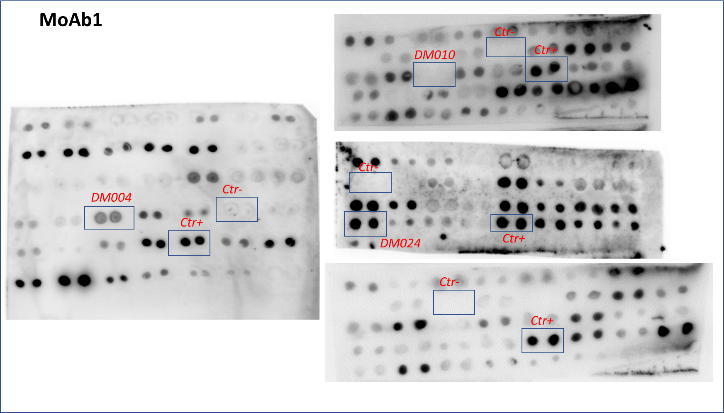


**Supplementary Figure S1**. Original, unprocessed images of the blots presented in Figure 1C.

Urinary LAM cut off [ng/mL]

**A**

**B**

**C**

**D**

**E**

**F**

2

4

6

8

10

0.0

0.2

0.4

0.6

Index

Probability Threshold

2

4

6

8

10

0.00

0.10

0.20

Index

2

4

6

8

10

0.0

0.2

0.4

Index

Classification Error

2

4

6

8

10

0.6

0.8

1.0

Index

ROC AUC

2

4

6

8

10

0.70

0.85

1.00

Index

Sensitivity

2

4

6

8

10

0.55

0.70

0.85

Index

Specificity

**Supplementary Figure S2**. Logistic regression and 10-fold cross validation studies were performed in order to define a urinary LAM cut off threshold. The 430 urine samples analyzed in this study were shuffled and split into 10 random groups. Ten combinations of train-test splits were obtained, whereby 9/10 groups were assigned to the train set and the remaining group was assigned to the test set without repetition. For ten times, a logistic regression model was trained on the train set and its prediction performance was assessed in the test set. The probability threshold, and consequently the LAM threshold, was chosen to yield a sensitivity of 0.90 and, at the same time, to maximize specificity. **A, B**: Estimates of probability threshold (mean 0.262, 95% CI: 0.256 - 0.268) and urinary LAM concentration cut off (0.080, 95% CI: 0.076 - 0.085) were stable across the cross validated sets. *The average cross validated probability cut off value of 0.262 for the logistic regression probability, equivalent to a urinary LAM concentration of 0.08 ng/mL, was chosen as a threshold for this study.* **C, D:** Out-of-sample area under the curve (0.903, 95% CI 0.883 - 0.923) and out-of-sample classification error (0.186, 95% CI: 0.156 - 0.216) values were concordant with in-sample values (AUC: 0.903, 95% CI: 0.900 - 0.906, *t-test p= 0.9807*; error: 0.182, 95% CI: 0.175 - 0.189, *t-test p = 0.8126*) and showed that the models generalized well within the random, shuffled test-train splits. **E, F**: As expected, in-sample sensitivity was tightly centered around 0.903 (95% CI 0.902 - 0.904), whereas in-sample specificity was 0.729 95% CI: 0.714 - 0.744. Out of sample sensitivity and specificity showed concordance (0.901, 95% CI: 0.858 - 0.944, 0.722, 95% CI: 0.646 - 0.799, respectively), further supporting robust performance of the models. In all figure panels, in-sample values are depicted in red and out of sample values are depicted in black.


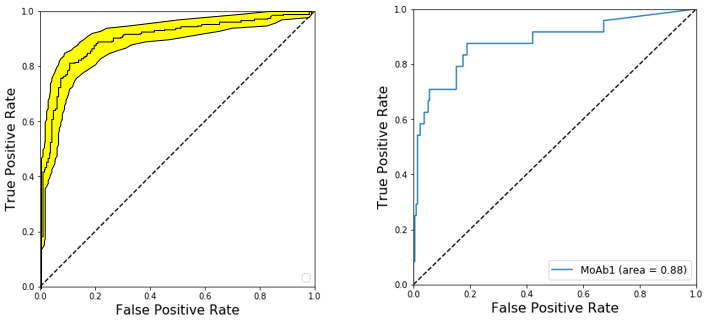


**Supplementary Figure S3**. MoAb1 retained high discriminatory power for TB positives versus negatives (AUC 0.88) in glycosuric patients.


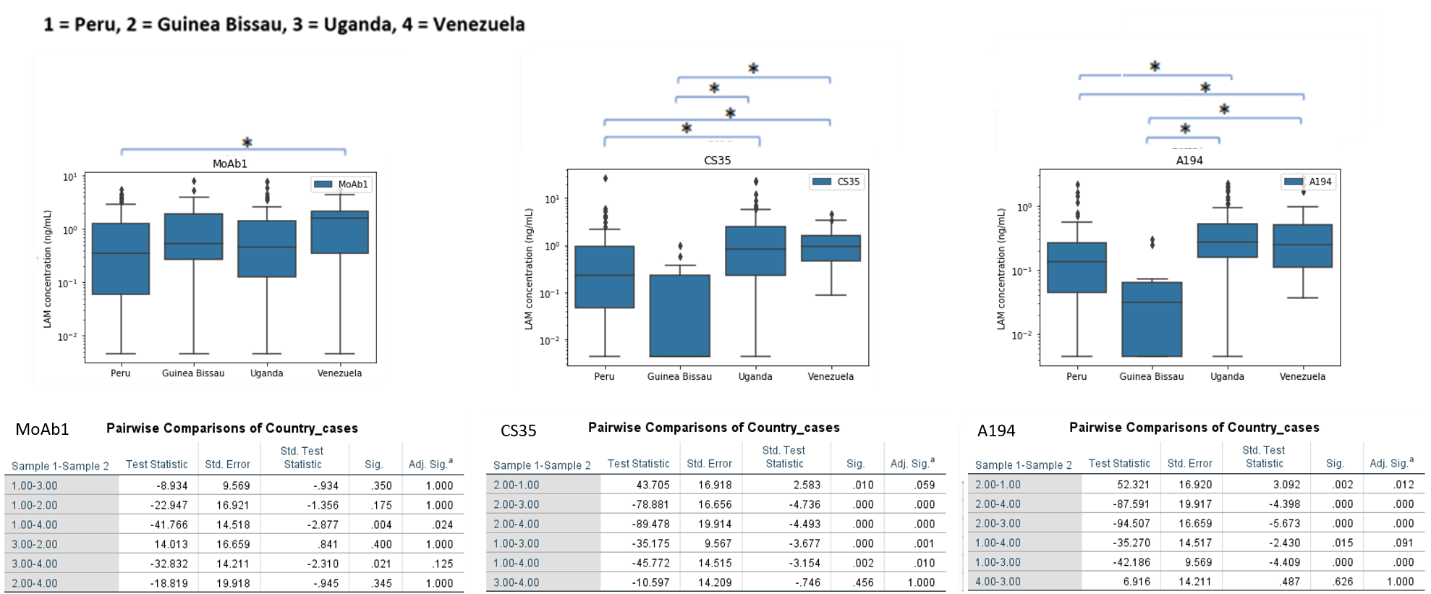


**Supplementary Figure S4**. Pairwise comparison of LAM concentration in TB positive patients grouped by country of origin. The highest LAM median concentration for MoAb1 and CS35 was observed in samples from Venezuela, while Uganda had the highest median concentration for A194. Guinea Bissau had the lowest LAM median concentration for CS35 and A194 while Peru had the lowest LAM median concentration for MoAb1.


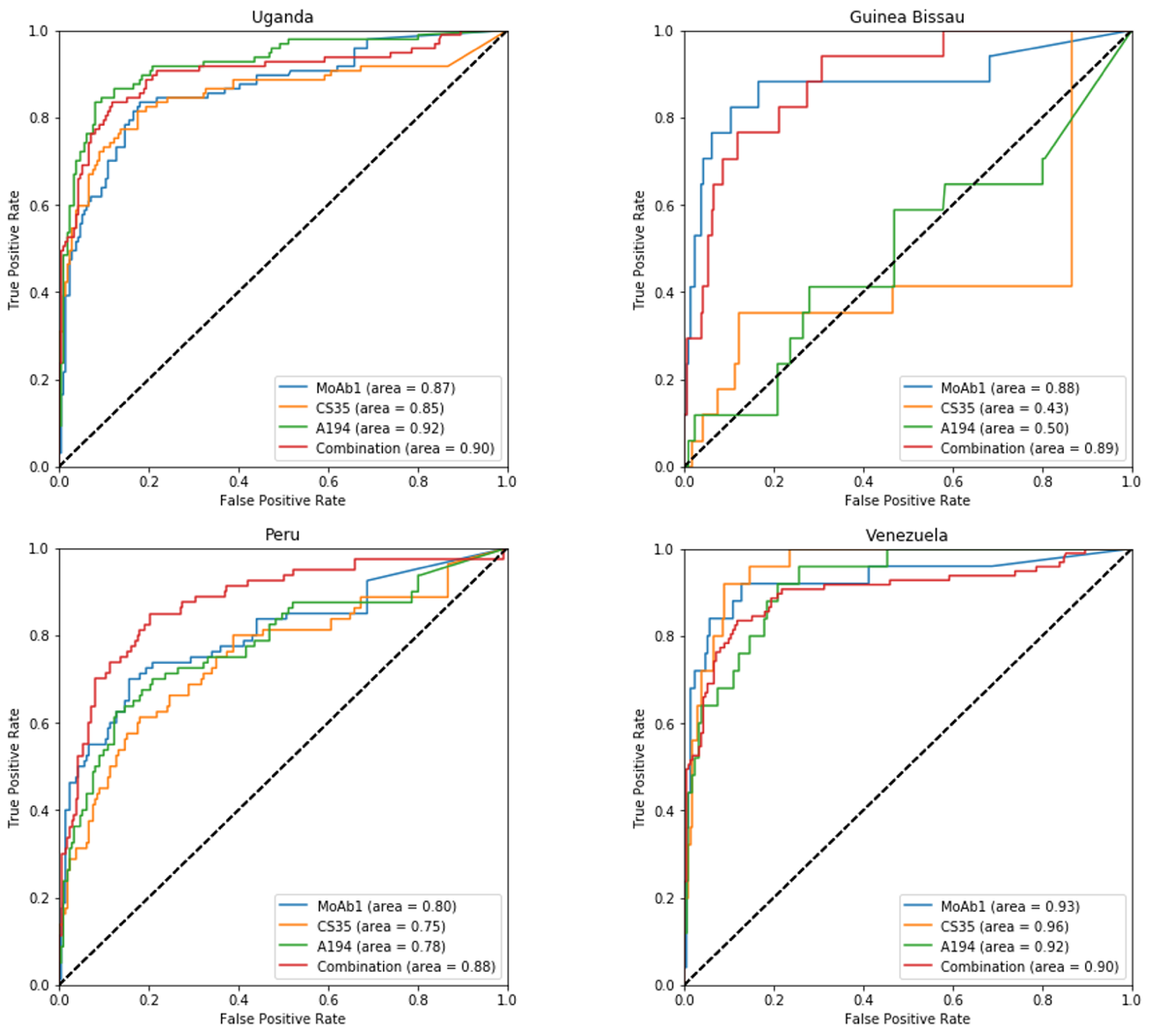


**Supplementary Figure S5**. Urinary LAM successfully distinguishes microbiologically confirmed untreated TB positives from TB negatives in each country cohort (Uganda, Guinea Bissau, Peru, Venezuela). While performance of each antibody varies by country, antibody combination increases overall performance.


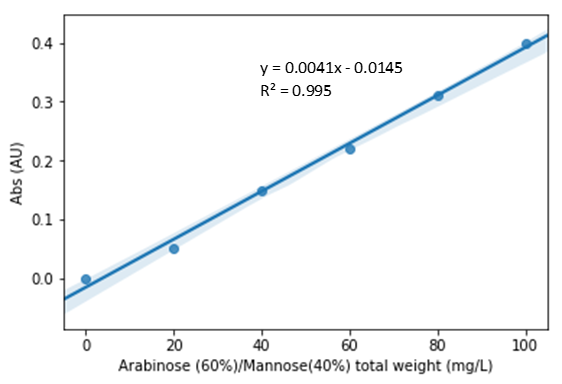


**Supplementary Figure S6**. Total saccharide concentration in the LAM reference standard (BEI Resources) was quantified by the Anthrone colorimetric method^29^. The assay was calibrated using a preparation composed of 60% Arabinose and 40% Mannose, according to established polysaccharide composition of the LAM arabinan and mannan domains^11,30,31^. LAM concentration measured using the Anthrone method was 0.398 mg/mL. Regression line with confidence interval (shaded area) shows linearity of the assay in the tested range.

# Supplementary Tables

**Supplementary Table 1**. List of antibodies tested in this study. MoAb1, CS35, A194 were selected based on their superior performance in urine spiked with increasing amounts of LAM calibrator (0, 0.6, 1.25, 2.5, 5 ng/mL).

| **Antibody** | **Expected reactivity** |
| --- | --- |
| p9045/p9276 | - |
| CS35 hu IgG | + |
| MoAb1 hu IgG | + |
| A194 hu IgM | + |
| A194 hu 2scFv (L4) | + |
| hu IgA neg control | - |
| hu IgM neg control | - |
| A194 hu IgG1 | + |
| A194 hu f(Ab)2 | + |
| A194 hu IgA | + |
| FIND28.ms IgG | + |
| CS35 ms.a1.f9 | + |

**Supplementary Table 2.** Results from urine dipstick analysis of sample cohort using Siemens Multistix 10SG Reagents Strips (pos=positive, neg=negative, QNS=Quantity Not Sufficient).

| **Patient ID** | **Urine #** | **Glucose** | **Bilirubin** | **Ketone** | **Specific Gravity** | **Blood** | **pH** | **Protein** | **Urobilinogen** | **Nitrite** | **Leukocytes** |
| --- | --- | --- | --- | --- | --- | --- | --- | --- | --- | --- | --- |
| O-DM001 | 1 | neg | neg | neg | 1.03 | neg | 6 | neg | 0.2 | neg | neg |
| O-DM002 | 2 | neg | neg | neg | 1.005 | neg | 5 | neg | 0.2 | neg | neg |
| O-DM004 | 3 | neg | neg | neg | 1.015 | neg | 6.5 | neg | 0.2 | neg | neg |
| O-DM014 | 4 | pos 4+ | neg | neg | 1.015 | neg | 6.5 | neg | 0.2 | neg | neg |
| O-DM003 | 5 | neg | moderate | neg | 1.02 | small | 5 | pos 1+ | 0.2 | neg | moderate |
| O-DM005 | 6 | neg | neg | neg | 1.02 | neg | 6.5 | neg | 0.2 | neg | neg |
| GMU65 | 9 | neg | neg | neg | 1.005 | neg | 6 | neg | 0.2 | neg | neg |
| O-DM006 | 11 | neg | neg | neg | 1.025 | neg | 6 | neg | 0.2 | neg | neg |
| O-DM008 | 12 | neg | neg | neg | 1.01 | neg | 6 | neg | 0.2 | neg | neg |
| O-DM009 | 13 | pos +4 | neg | large | 1.015 | neg | 6 | trace | 1 | neg | neg |
| O-DM010 | 14 | neg | neg | neg | 1.02 | neg | 7 | neg | 0.2 | neg | neg |
| O-DM027 | 16 | neg | neg | neg | 1.015 | neg | 8 | neg | 0.2 | pos | neg |
| GMU82 | 20 | neg | neg | neg | 1.02 | neg | 6.5 | neg | 0.2 | neg | large |
| O-DM012 | 22 | neg | neg | neg | 1.02 | small | 7 | pos +1 | 0.2 | neg | neg |
| O-DM013 | 23 | neg | neg | neg | 1.01 | neg | 6 | neg | 0.2 | neg | neg |
| O-DM014 | 24 | neg | neg | neg | 1.01 | small | 6 | neg | 0.2 | neg | neg |
| GMU83 | 29 | neg | neg | neg | 1.02 | neg | 5 | neg | 0.2 | neg | neg |
| O-DM015 | 31 | neg | neg | neg | 1.01 | small | 6 | neg | 0.2 | neg | neg |
| O-DM016 | 32 | neg | small | neg | 1.02 | neg | 6.5 | neg | 0.2 | neg | neg |
| O-DM017 | 33 | neg | neg | neg | 1.02 | trace | 6.5 | neg | 0.2 | neg | trace |
| O-DM018 | 34 | pos +4 | neg | neg | 1.015 | neg | 6.5 | neg | 0.2 | neg | neg |
| O-DM019 | 41 | trace | neg | neg | 1.01 | neg | 7.5 | neg | 0.2 | neg | neg |
| O-DM020 | 42 | neg | moderate | neg | 1.015 | large | 6 | pos 1+ | 2 | neg | large |
| O-DM021 | 43 | neg | neg | neg | 1.01 | neg | 6 | neg | 0.2 | neg | neg |
| O-DM011 | 44 | trace | large | trace | 1.02 | neg | 7 | neg | 4 | neg | neg |
| O-DM022 | 45 | neg | neg | neg | 1.01 | neg | 6 | pos 2+ | 0.2 | neg | neg |
| O-DM023 | 51 | neg | neg | neg | 1.015 | neg | 6 | neg | 0.2 | neg | neg |
| O-DM024 | 52 | neg | neg | neg | 1.02 | trace | 6.5 | trace | 0.2 | neg | neg |
| O-DM025 | 53 | neg | neg | neg | 1.015 | neg | 7 | neg | 0.2 | neg | neg |
| O-DM026 | 54 | pos 4+ | neg | trace | 1.01 | neg | 6.5 | neg | 0.2 | neg | neg |
| O-DM028 | 61 | neg | neg | neg | 1.02 | neg | 6.5 | neg | 0.2 | neg | neg |
| O-DM029 | 62 | neg | neg | neg | 1.025 | neg | 6 | neg | 0.2 | neg | neg |
| O-DM030 | 63 | pos 1+ | large | neg | 1.02 | neg | 6 | neg | 2 | neg | neg |
| O-DM031 | 64 | neg | neg | neg | 1.015 | neg | 6 | neg | 0.2 | neg | neg |
| O-DM032 | 71 | neg | neg | neg | 1.005 | neg | 7 | neg | 0.2 | neg | neg |
| O-DM033 | 72 | neg | neg | neg | 1.015 | neg | 6.5 | neg | 0.2 | neg | neg |
| O-HC501 | 73 | neg | neg | neg | 1.02 | neg | 6.5 | neg | 0.2 | neg | neg |
| GMU80 | 79 | neg | neg | neg | 1.015 | neg | 6 | trace | 0.2 | neg | neg |
| O-HC502 | 81 | neg | neg | neg | 1.015 | neg | 8.5 | neg | 0.2 | neg | neg |
| O-HC503 | 82 | neg | neg | neg | 1.01 | moderate | 8 | trace | 0.2 | neg | trace |
| O-HC504 | 83 | pos 4+ | neg | large | 1.015 | neg | 6.5 | trace | 0.2 | neg | neg |
| O-HC505 | 85 | neg | QNS | QNS | QNS | neg | QNS | QNS | QNS | QNS | neg |
| R-DM024 | 86 | neg | QNS | QNS | QNS | QNS | QNS | QNS | QNS | QNS | QNS |
| R-SC008 | 87 | neg | QNS | QNS | QNS | QNS | QNS | QNS | QNS | QNS | QNS |
| R-SC018 | 88 | neg | neg | neg | 1.015 | neg | 6 | neg | 0.2 | neg | neg |
| R-TC002 | 89 | neg | neg | neg | 1.015 | trace | 7.5 | trace | 0.2 | neg | large |
| GMU81 | 89 | neg | neg | neg | 1.015 | trace | 7.5 | trace | 0.2 | neg | large |
| U-NTT197 | 91 | neg | neg | neg | 1.01 | neg | 6 | neg | 0.2 | neg | large |
| U-NTT162Â | 92 | neg | neg | neg | 1.01 | neg | 7.5 | trace | 0.2 | neg | moderate |
| U-NTT192 | 93 | neg | neg | neg | 1.015 | neg | 6 | neg | 0.2 | neg | trace |
| GB-7AÂ | 94 | pos 1+ | neg | neg | 1.015 | trace | 6 | trace | 0.2 | neg | trace |
| GB-1AÂ | 95 | neg | moderate | neg | 1.015 | neg | 8 | trace | 0.2 | neg | neg |
| GB-13AÂ | 96 | neg | small | neg | 1.01 | neg | 7 | trace | 0.2 | neg | trace |
| U-NTT155 | 101 | neg | neg | neg | 1.01 | neg | 8.5 | neg | 0.2 | neg | neg |
| GB-8AÂ | 102 | neg | moderate | neg | 1.025 | neg | 6 | pos 2+ | 0.2 | neg | trace |
| U-NTT173Â | 103 | neg | neg | neg | 1.01 | neg | 6.5 | neg | 0.2 | neg | neg |
| GB-10AÂ | 104 | neg | neg | neg | 1.01 | neg | 6 | neg | 0.2 | neg | neg |
| GB-2A | 104 | neg | neg | neg | 1.01 | neg | 6 | neg | 0.2 | neg | neg |
| GB-3AÂ | 105 | neg | neg | neg | 1.015 | neg | 6.5 | pos 1+ | 0.2 | neg | neg |
| GB-14AÂ | 106 | pos 1+ | small | small | 1.015 | moderate | 6.5 | pos 3+ | 0.2 | neg | large |
| U-NTT179 | 111 | neg | QNS | QNS | QNS | QNS | QNS | QNS | QNS | QNS | QNS |
| U-NTT181Â | 112 | neg | QNS | QNS | QNS | QNS | QNS | QNS | QNS | QNS | QNS |
| GB-5AÂ | 113 | pos 1+ | moderate | neg | 1.015 | neg | 6.5 | trace | 0.2 | neg | neg |
| GB-6AÂ | 114 | trace | small | trace | 1.01 | neg | 8 | pos 1+ | 0.2 | neg | neg |
| GB-17AÂ | 115 | neg | neg | neg | 1.015 | trace | 6 | pos 2+ | 0.2 | neg | neg |
| GB-18AÂ | 116 | neg | moderate | neg | 1.02 | neg | 6.5 | pos 1+ | 0.2 | neg | neg |
| GB-4A | 121 | neg | neg | neg | 1.01 | neg | 6 | neg | 0.2 | neg | neg |
| GB-15AÂ | 122 | pos 1+ | neg | neg | 1.005 | neg | 8 | neg | 0.2 | neg | neg |
| GB-16AÂ | 123 | pos 4+ | neg | neg | 1.01 | neg | 7.5 | neg | 0.2 | neg | neg |
| V-28Â | 124 | neg | QNS | QNS | QNS | QNS | QNS | QNS | QNS | QNS | QNS |
| V-30Â | 125 | neg | QNS | QNS | QNS | QNS | QNS | QNS | QNS | QNS | QNS |
| V-31 | 126 | neg | QNS | QNS | QNS | QNS | QNS | QNS | QNS | QNS | QNS |
| L35 | 127 | neg | neg | neg | 1.01 | neg | 8.5 | neg | 0.2 | neg | neg |
| L62 | 128 | neg | neg | neg | 1.015 | neg | 6 | neg | 0.2 | neg | neg |
| V-41Â | 131 | neg | QNS | QNS | QNS | QNS | QNS | QNS | QNS | QNS | QNS |
| V-44Â | 132 | neg | QNS | QNS | QNS | QNS | QNS | QNS | QNS | QNS | QNS |
| V-46Â | 133 | neg | QNS | QNS | QNS | QNS | QNS | QNS | QNS | QNS | QNS |
| V-50Â | 134 | pos 4+ | QNS | QNS | QNS | QNS | QNS | QNS | QNS | QNS | QNS |
| V-51Â | 136 | neg | QNS | QNS | QNS | QNS | QNS | QNS | QNS | QNS | QNS |
| V-33Â | 141 | neg | QNS | QNS | QNS | QNS | QNS | QNS | QNS | QNS | QNS |
| V-34Â | 142 | neg | QNS | QNS | QNS | QNS | QNS | QNS | QNS | QNS | QNS |
| V-36Â | 143 | neg | QNS | QNS | QNS | QNS | QNS | QNS | QNS | QNS | QNS |
| V-38Â | 144 | neg | neg | neg | 1.02 | neg | 6 | pos 1+ | 0.2 | neg | neg |
| V-39Â | 145 | neg | QNS | QNS | QNS | QNS | QNS | QNS | QNS | QNS | QNS |
| V-40Â | 146 | neg | QNS | QNS | QNS | QNS | QNS | QNS | QNS | QNS | QNS |
| L7 | 147 | neg | neg | neg | 1.015 | neg | 8.5 | neg | 0.2 | neg | neg |
| L8 | 148 | neg | neg | large | 1.015 | neg | 7.5 | neg | 0.2 | neg | neg |
| V-53Â | 151 | neg | neg | neg | 1.01 | neg | 6 | trace | 0.2 | neg | large |
| V-54Â | 152 | neg | neg | neg | 1.01 | trace | 6 | neg | 0.2 | neg | neg |
| V-55 | 153 | neg | neg | neg | 1.015 | moderate | 5 | trace | 0.2 | neg | neg |
| V-57 | 154 | neg | neg | neg | 1.015 | neg | 6 | neg | 0.2 | neg | neg |
| V-58Â | 155 | neg | neg | neg | 1.03 | neg | 5 | trace | 0.2 | neg | neg |
| V-59Â | 156 | neg | neg | neg | 1.01 | neg | 6 | neg | 0.2 | neg | neg |
| L9 | 157 | neg | neg | neg | 1.01 | neg | 8 | neg | 0.2 | neg | neg |
| V-60Â | 161 | neg | neg | neg | 1.025 | moderate | 6 | trace | 0.2 | neg | neg |
| V-64Â | 164 | neg | neg | neg | 1.015 | large | 6 | pos 1+ | 0.2 | neg | neg |
| V-65 | 165 | neg | neg | neg | 1.015 | neg | 6.5 | neg | 0.2 | neg | neg |
| GB-9AÂ | 166 | neg | neg | neg | 1.02 | neg | 6.5 | neg | 0.2 | neg | neg |
| GB-12AÂ | 171 | neg | neg | neg | 1.01 | neg | 8 | neg | 0.2 | neg | neg |
| O-HC506 | 172 | neg | neg | neg | 1.01 | neg | 6.5 | neg | 0.2 | neg | neg |
| O-HC539 | 172 | neg | neg | neg | 1.01 | neg | 6.5 | neg | 0.2 | neg | neg |
| O-HC507 | 173 | neg | neg | neg | 1.015 | neg | 6 | neg | 0.2 | neg | neg |
| O-HC509 | 174 | neg | neg | neg | 1.015 | small | 6 | trace | 0.2 | neg | neg |
| O-HC510 | 175 | neg | large | neg | 1.015 | neg | 6.5 | neg | 0.2 | neg | neg |
| O-HC511 | 175 | neg | large | neg | 1.015 | neg | 6.5 | neg | 0.2 | neg | neg |
| O-HC515 | 175 | neg | large | neg | 1.015 | neg | 6.5 | neg | 0.2 | neg | neg |
| O-HC512 | 176 | neg | neg | neg | 1.01 | neg | 8 | neg | 0.2 | neg | neg |
| O-HC513 | 181 | neg | neg | neg | 1.015 | neg | 8.5 | neg | 0.2 | neg | neg |
| O-HC514 | 182 | pos 4+ | neg | neg | 1.005 | neg | 6 | neg | 0.2 | neg | neg |
| O-HC514 | 182 | pos 4+ | neg | neg | 1.005 | neg | 6 | neg | 0.2 | neg | neg |
| O-HC516 | 183 | trace | neg | neg | 1.005 | neg | 8 | neg | 0.2 | neg | neg |
| O-HC517 | 184 | neg | small | neg | 1.015 | neg | 6 | neg | 0.2 | neg | neg |
| O-HC528ÃÂ ÃÂ | 184 | neg | small | neg | 1.015 | neg | 6 | neg | 0.2 | neg | neg |
| O-HC518 | 185 | neg | neg | moderate | 1.015 | trace | 6 | trace | 0.2 | neg | moderate |
| O-HC520 | 191 | trace | neg | neg | 1.015 | neg | 7 | neg | 0.2 | neg | neg |
| O-HC521 | 192 | neg | moderate | neg | 1.02 | neg | 6 | trace | 0.2 | neg | moderate |
| O-HC522 | 193 | neg | neg | neg | 1.015 | neg | 8 | trace | 0.2 | neg | neg |
| O-HC523 | 194 | neg | neg | neg | 1.02 | moderate | 7 | trace | 0.2 | neg | large |
| O-HC524 | 195 | neg | neg | neg | 1.02 | trace | 7 | trace | 0.2 | neg | neg |
| O-HC525 | 196 | pos 3+ | small | neg | 1.015 | moderate | 6.5 | pos 1+ | 0.2 | neg | neg |
| O-HC526 | 201 | neg | small | neg | 1.015 | moderate | 6 | trace | 0.2 | neg | neg |
| O-HC529 | 203 | neg | moderate | neg | 1.015 | neg | 8 | neg | 0.2 | neg | neg |
| O-HC530 | 204 | neg | small | neg | 1.02 | large | 7 | trace | 0.2 | neg | neg |
| UW-117 | 207 | neg | neg | neg | 1.015 | large | 6 | neg | 0.2 | neg | trace |
| UW-122 | 208 | neg | neg | neg | 1.01 | moderate | 6 | pos 1+ | 0.2 | neg | trace |
| O-HC531 | 211 | neg | neg | neg | 1.01 | neg | 8.5 | neg | 0.2 | neg | neg |
| O-HC532 | 212 | neg | small | neg | 1.015 | neg | 5 | trace | 0.2 | neg | neg |
| O-HC533 | 213 | neg | neg | neg | 1.02 | neg | 6.5 | trace | 0.2 | neg | neg |
| O-HC534 | 214 | neg | large | neg | 1.02 | neg | 6 | pos 3+ | 0.2 | neg | neg |
| O-HC535 | 215 | neg | neg | neg | 1.02 | neg | 8 | neg | 0.2 | neg | neg |
| O-HC536 | 216 | neg | neg | neg | 1.01 | neg | 7.5 | trace | 0.2 | neg | neg |
| O-HC537 | 217 | pos 4+ | neg | moderate | 1.02 | trace | 6 | pos 1+ | 0.2 | neg | neg |
| O-HC538 | 218 | neg | neg | neg | 1.015 | neg | 6 | neg | 0.2 | neg | neg |
| O-HC540 | 219 | neg | moderate | neg | 1.03 | small | 6 | neg | 0.2 | neg | small |
| O-HC541 | 220 | pos 4+ | neg | neg | 1.02 | neg | 6 | neg | 0.2 | neg | neg |
| O-HC542 | 221 | neg | neg | neg | 1.01 | neg | 6 | trace | 0.2 | neg | neg |
| UW-119 | 221 | neg | neg | neg | 1.01 | neg | 6 | trace | 0.2 | neg | neg |
| UW-120 | 222 | neg | neg | neg | 1.01 | neg | 6 | neg | 0.2 | neg | neg |
| UW-124 | 223 | neg | neg | neg | 1.015 | neg | 7 | neg | 0.2 | neg | neg |
| UW-476 | 224 | neg | neg | neg | 1.005 | neg | 7 | neg | 0.2 | neg | neg |
| UW-736 | 225 | neg | neg | neg | 1.015 | large | 6 | trace | 0.2 | neg | small |
| R-DM013 | 226 | neg | neg | neg | 1.01 | moderate | 7 | trace | 0.2 | neg | large |
| UW-738 | 226 | neg | neg | neg | 1.01 | moderate | 7 | trace | 0.2 | neg | large |
| UW-739 | 227 | neg | neg | neg | 1.01 | neg | 7.5 | neg | 0.2 | neg | neg |
| UW-740 | 228 | neg | neg | neg | 1.015 | small | 6 | trace | 0.2 | neg | large |
| R-DM020 | 229 | neg | neg | neg | 1.01 | neg | 8.5 | neg | 0.2 | neg | neg |
| UW-477 | 229 | neg | neg | neg | 1.01 | neg | 8.5 | neg | 0.2 | neg | neg |
| UW-482 | 230 | neg | neg | neg | 1.02 | large | 6 | pos 1+ | 0.2 | neg | neg |
| R-DM022 | 231 | neg | neg | neg | 1.02 | neg | 6 | neg | 0.2 | neg | moderate |
| UW-741 | 234 | neg | neg | neg | 1.02 | large | 6 | neg | 0.2 | neg | small |
| UW-743 | 235 | neg | neg | neg | 1.01 | neg | 8.5 | neg | 0.2 | neg | neg |
| UW-744 | 236 | neg | neg | neg | 1.01 | neg | 8 | neg | 0.2 | neg | neg |
| UW-745 | 237 | neg | neg | neg | 1.015 | neg | 7.5 | trace | 0.2 | neg | neg |
| UW-485 | 238 | neg | moderate | neg | 1.01 | neg | 6 | pos 1+ | 1 | neg | neg |
| UW-491 | 239 | neg | neg | neg | 1.01 | neg | 6.5 | neg | 0.2 | neg | neg |
| UW-758 | 242 | neg | neg | neg | 1.015 | neg | 6.5 | neg | 0.2 | neg | neg |
| UW-763 | 243 | neg | neg | neg | 1.015 | neg | 7 | neg | 0.2 | neg | neg |
| UW-766 | 244 | neg | neg | neg | 1.015 | neg | 8.5 | neg | 0.2 | neg | neg |
| UW-767 | 245 | neg | neg | neg | 1.02 | neg | 7 | neg | 0.2 | neg | neg |
| UW-501 | 246 | neg | neg | neg | 1.01 | neg | 8 | neg | 0.2 | neg | neg |
| UW-504 | 247 | neg | neg | neg | 1.01 | neg | 7 | trace | 0.2 | neg | neg |
| UW-507 | 247 | neg | neg | neg | 1.01 | neg | 7 | trace | 0.2 | neg | neg |
| UW-768 | 250 | neg | neg | neg | 1.01 | neg | 8.5 | neg | 0.2 | neg | neg |
| UW-771 | 251 | neg | neg | neg | 1.015 | neg | 7.5 | trace | 0.2 | neg | neg |
| UW-774 | 252 | neg | neg | neg | 1.015 | neg | 8 | neg | 0.2 | neg | neg |
| UW-783 | 253 | neg | neg | neg | 1.01 | neg | 7 | neg | 0.2 | neg | neg |
| UW-531 | 254 | neg | neg | neg | 1.01 | neg | 8.5 | neg | 0.2 | neg | neg |
| UW-578 | 255 | neg | neg | neg | 1.005 | trace | 6 | trace | 0.2 | neg | neg |
| UW-784 | 258 | neg | neg | neg | 1.01 | neg | 8 | neg | 0.2 | neg | neg |
| UW-502 | 259 | neg | neg | neg | 1.005 | neg | 7.5 | neg | 0.2 | neg | moderate |
| UW-505 | 259 | neg | neg | neg | 1.005 | neg | 7.5 | neg | 0.2 | neg | moderate |
| UW-788 | 260 | neg | neg | neg | 1.015 | neg | 6 | neg | 0.2 | neg | neg |
| UW-794 | 261 | neg | neg | neg | 1.01 | small | 6 | neg | 0.2 | neg | neg |
| UW-611 | 262 | neg | neg | neg | 1.015 | neg | 7 | pos 1+ | 0.2 | neg | neg |
| UW-649 | 263 | neg | large | neg | 1.01 | neg | 7.5 | pos 1+ | 4 | neg | neg |
| UW-799 | 266 | neg | neg | neg | 1.005 | neg | 8 | neg | 0.2 | neg | neg |
| UW-803 | 267 | neg | small | neg | 1.01 | neg | 7 | trace | 0.2 | neg | neg |
| UW-807 | 268 | neg | small | neg | 1.01 | neg | 8.5 | trace | 0.2 | neg | neg |
| UW-812 | 269 | neg | neg | neg | 1.01 | small | 8.5 | trace | 0.2 | neg | neg |
| UW-656 | 270 | neg | neg | neg | 1.005 | neg | 7 | neg | 0.2 | neg | neg |
| UW-667 | 271 | neg | neg | neg | 1.015 | neg | 6 | pos 1+ | 0.2 | neg | neg |
| UW-814 | 274 | neg | neg | neg | 1.01 | neg | 8.5 | neg | 0.2 | neg | neg |
| UW-818 | 275 | neg | neg | neg | 1.015 | neg | 7.5 | trace | 0.2 | neg | neg |
| UW-820 | 276 | neg | neg | neg | 1.01 | neg | 8.5 | neg | 0.2 | neg | neg |
| UW-823 | 277 | neg | small | neg | 1.01 | neg | 8.5 | trace | 0.2 | neg | neg |
| UW-668 | 278 | neg | neg | neg | 1.01 | neg | 8 | neg | 0.2 | neg | neg |
| UW-685 | 279 | neg | neg | neg | 1.005 | small | 6 | trace | 0.2 | neg | large |
| UW-831 | 283 | neg | neg | neg | 1.02 | neg | 6 | trace | 0.2 | neg | neg |
| UW-706 | 290 | neg | neg | neg | 1.01 | neg | 8 | neg | 0.2 | neg | neg |
| UW-707 | 291 | neg | neg | neg | 1.025 | neg | 6 | trace | 0.2 | neg | neg |
| UW-718 | 292 | neg | neg | neg | 1.01 | neg | 7 | trace | 0.2 | neg | moderate |
| UW-721 | 293 | neg | small | neg | 1.015 | moderate | 7.5 | pos 1+ | 0.2 | neg | trace |
| UW-722 | 300 | neg | neg | neg | 1.01 | neg | 8.5 | neg | 1 | neg | neg |
| UW-733 | 301 | neg | neg | small | 1.01 | neg | 7 | pos 1+ | 0.2 | neg | neg |
| UW-735 | 302 | neg | moderate | neg | 1.02 | large | 6 | pos 2+ | 0.2 | neg | large |
| UW-742 | 303 | neg | small | neg | 1.015 | neg | 6 | pos 1+ | 0.2 | neg | neg |
| UW-757 | 310 | neg | neg | neg | 1.01 | neg | 8 | neg | 0.2 | neg | moderate |
| UW-764 | 311 | neg | neg | neg | 1.01 | neg | 8.5 | neg | 0.2 | neg | large |
| UW-775 | 312 | neg | small | neg | 1.015 | neg | 6 | pos 1+ | 0.2 | neg | neg |
| UW-793 | 313 | neg | small | neg | 1.015 | neg | 6 | trace | 0.2 | neg | trace |
| UW-805 | 320 | neg | neg | neg | 1.015 | neg | 6 | pos 1+ | 0.2 | neg | neg |
| UW-808 | 321 | neg | small | neg | 1.01 | neg | 8.5 | trace | 0.2 | neg | neg |
| UW-811 | 322 | neg | neg | neg | 1.01 | neg | 8.5 | trace | 0.2 | neg | neg |
| UW-817 | 323 | neg | neg | neg | 1.015 | large | 6.5 | pos 2+ | 0.2 | neg | trace |
| UW-845 | 330 | trace | small | neg | 1.02 | neg | 6 | pos 3+ | 0.2 | neg | trace |
| UW-851 | 331 | neg | neg | neg | 1.02 | neg | 7.5 | trace | 0.2 | neg | neg |
| UW-871 | 332 | neg | small | neg | 1.015 | moderate | 6.5 | pos 3+ | 0.2 | neg | neg |
| UW-876 | 333 | neg | small | neg | 1.015 | neg | 6.5 | pos 2+ | 0.2 | neg | neg |
| UW-533 | 388 | neg | neg | neg | 1.01 | small | 6.5 | trace | 0.2 | pos | moderate |
| GMU16 | 390 | neg | neg | neg | 1.015 | neg | 7 | neg | 0.2 | neg | neg |
| GMU17 | 392 | neg | neg | neg | 1.02 | neg | 7.5 | neg | 0.2 | neg | trace |
| UW-750 | 393 | neg | neg | neg | 1.025 | moderate | 6 | trace | 0.2 | neg | moderate |
| GMU19 | 395 | neg | neg | neg | 1.025 | neg | 6 | neg | 0.2 | neg | neg |
| GMU20 | 396 | neg | neg | neg | 1.015 | neg | 6.5 | neg | 0.2 | neg | neg |
| GMU21 | 398 | neg | neg | neg | 1.01 | neg | 8.5 | trace | 0.2 | neg | trace |
| GMU22 | 400 | neg | neg | neg | 1.01 | neg | 6.5 | neg | 0.2 | neg | neg |
| GMU23 | 402 | neg | neg | neg | 1.015 | neg | 7.5 | trace | 0.2 | neg | neg |
| GMU24 | 403 | neg | neg | neg | 1.02 | neg | 6 | trace | 0.2 | neg | neg |
| UW-543 | 404 | neg | neg | neg | 1.01 | neg | 7.5 | neg | 0.2 | neg | neg |
| UW-500 | 407 | neg | neg | neg | 1.01 | neg | 6.5 | neg | 0.2 | neg | neg |
| GMU1 | 408 | neg | neg | neg | 1.015 | large | 6 | trace | 0.2 | neg | trace |
| GMU84 | 408 | neg | neg | neg | 1.015 | large | 6 | trace | 0.2 | neg | trace |
| GMU2 | 409 | neg | neg | neg | 1.01 | neg | 7 | neg | 0.2 | neg | neg |
| GMU85 | 409 | neg | neg | neg | 1.01 | neg | 7 | neg | 0.2 | neg | neg |
| GMU3 | 410 | neg | neg | neg | 1.01 | neg | 7 | trace | 0.2 | neg | moderate |
| GMU86 | 410 | neg | neg | neg | 1.01 | neg | 7 | trace | 0.2 | neg | moderate |
| GMU4 | 411 | neg | neg | neg | 1.015 | neg | 7.5 | trace | 0.2 | neg | neg |
| GMU87 | 411 | neg | neg | neg | 1.015 | neg | 7.5 | trace | 0.2 | neg | neg |
| GMU5 | 412 | neg | neg | neg | 1.015 | neg | 7 | trace | 0.2 | neg | moderate |
| GMU88 | 412 | neg | neg | neg | 1.015 | neg | 7 | trace | 0.2 | neg | moderate |
| GMU6 | 413 | neg | neg | neg | 1.015 | large | 6.5 | trace | 0.2 | neg | small |
| GMU89 | 413 | neg | neg | neg | 1.015 | large | 6.5 | trace | 0.2 | neg | small |
| GMU7 | 414 | neg | neg | neg | 1.015 | neg | 6 | neg | 0.2 | neg | neg |
| GMU90 | 414 | neg | neg | neg | 1.015 | neg | 6 | neg | 0.2 | neg | neg |
| GMU8 | 417 | neg | neg | neg | 1.015 | neg | 6.5 | neg | 0.2 | neg | neg |
| GMU91 | 417 | neg | neg | neg | 1.015 | neg | 6.5 | neg | 0.2 | neg | neg |
| GMU9 | 420 | neg | neg | neg | 1.015 | neg | 6.5 | neg | 0.2 | neg | neg |
| GMU92 | 420 | neg | neg | neg | 1.015 | neg | 6.5 | neg | 0.2 | neg | neg |
| GMU10 | 422 | neg | neg | neg | 1.01 | moderate | 8.5 | trace | 0.2 | neg | moderate |
| GMU93 | 422 | neg | neg | neg | 1.01 | moderate | 8.5 | trace | 0.2 | neg | moderate |
| UW-542 | 427 | neg | neg | neg | 1.02 | moderate | 6 | trace | 0.2 | neg | small |
| GMU94 | 427 | neg | neg | neg | 1.02 | moderate | 6 | trace | 0.2 | neg | small |
| GMU12 | 428 | neg | neg | neg | 1.01 | neg | 7.5 | neg | 0.2 | neg | trace |
| GMU95 | 428 | neg | neg | neg | 1.01 | neg | 7.5 | neg | 0.2 | neg | trace |
| GMU13 | 430 | neg | neg | neg | 1.015 | large | 6 | trace | 0.2 | neg | small |
| GMU96 | 430 | neg | neg | neg | 1.015 | large | 6 | trace | 0.2 | neg | small |
| GMU14 | 433 | neg | neg | neg | 1.015 | neg | 6.5 | trace | 0.2 | neg | trace |
| GMU97 | 433 | neg | neg | neg | 1.015 | neg | 6.5 | trace | 0.2 | neg | trace |
| GMU30 | 445 | neg | neg | neg | 1.01 | neg | 7.5 | trace | 0.2 | neg | neg |
| GMU31 | 445 | neg | neg | neg | 1.01 | neg | 7.5 | trace | 0.2 | neg | neg |
| GMU32 | 445 | neg | neg | neg | 1.01 | neg | 7.5 | trace | 0.2 | neg | neg |
| GMU37 | 446 | neg | neg | neg | 1.005 | neg | 8.5 | neg | 0.2 | neg | neg |
| GMU38 | 447 | neg | neg | neg | 1.01 | neg | 8.5 | neg | 0.2 | neg | neg |
| GMU39 | 448 | neg | neg | neg | 1.015 | neg | 6.5 | neg | 0.2 | neg | neg |
| GMU40 | 449 | neg | neg | neg | 1.01 | neg | 7.5 | neg | 0.2 | neg | neg |
| GMU41 | 450 | neg | neg | neg | 1.005 | neg | 8.5 | neg | 0.2 | neg | neg |
| GMU29 | 451 | neg | neg | neg | 1.01 | small | 8 | neg | 0.2 | neg | neg |
| GMU43 | 452 | neg | neg | neg | 1.015 | neg | 6 | neg | 0.2 | neg | neg |
| GMU45 | 453 | neg | neg | neg | 1.01 | neg | 8.5 | neg | 0.2 | neg | neg |
| GMU47 | 454 | neg | neg | neg | 1.015 | neg | 6 | neg | 0.2 | neg | neg |
| GMU46 | 455 | neg | neg | neg | 1.01 | neg | 6.5 | neg | 0.2 | neg | neg |
| GMU44 | 456 | neg | neg | neg | 1.01 | neg | 7 | neg | 0.2 | neg | neg |
| GMU58 | 457 | neg | neg | moderate | 1.02 | neg | 6 | trace | 0.2 | neg | small |
| GMU59 | 457 | neg | neg | moderate | 1.02 | neg | 6 | trace | 0.2 | neg | small |
| GMU60 | 457 | neg | neg | moderate | 1.02 | neg | 6 | trace | 0.2 | neg | small |
| GMU36 | 458 | neg | neg | neg | 1.015 | neg | 6 | neg | 0.2 | neg | neg |
| GMU48 | 459 | neg | neg | neg | 1.015 | neg | 6 | neg | 0.2 | neg | neg |
| UW-543 | 460 | neg | neg | neg | 1.01 | large | 7.5 | neg | 0.2 | neg | trace |
| UW-734 | 460 | neg | neg | neg | 1.01 | large | 7.5 | neg | 0.2 | neg | trace |
| GMU53 | 460 | neg | neg | neg | 1.01 | large | 7.5 | neg | 0.2 | neg | trace |
| GMU49 | 461 | neg | neg | neg | 1.015 | neg | 6 | trace | 0.2 | neg | neg |
| GMU54 | 462 | neg | neg | trace | 1.02 | neg | 6 | trace | 0.2 | neg | neg |
| GMU50 | 463 | neg | neg | neg | 1.015 | neg | 7 | trace | 0.2 | neg | neg |
| GMU55 | 464 | neg | neg | trace | 1.015 | neg | 6.5 | trace | 0.2 | neg | neg |
| GMU56 | 464 | neg | neg | trace | 1.015 | neg | 6.5 | trace | 0.2 | neg | neg |
| GMU57 | 464 | neg | neg | trace | 1.015 | neg | 6.5 | trace | 0.2 | neg | neg |
| GMU33 | 465 | neg | neg | neg | 1.01 | neg | 7.5 | neg | 0.2 | neg | neg |
| GMU34 | 465 | neg | neg | neg | 1.01 | neg | 7.5 | neg | 0.2 | neg | neg |
| GMU35 | 465 | neg | neg | neg | 1.01 | neg | 7.5 | neg | 0.2 | neg | neg |
| GMU42 | 466 | neg | neg | neg | 1.01 | neg | 6.5 | neg | 0.2 | neg | neg |
| GMU61 | 467 | neg | neg | neg | 1.01 | neg | 8 | trace | 0.2 | neg | neg |
| GMU62 | 467 | neg | neg | neg | 1.01 | neg | 8 | trace | 0.2 | neg | neg |
| GMU63 | 467 | neg | neg | neg | 1.01 | neg | 8 | trace | 0.2 | neg | neg |
| GMU64 | 468 | neg | neg | neg | 1.005 | neg | 7.5 | neg | 0.2 | neg | neg |
| GMU66 | 469 | neg | neg | neg | 1.02 | neg | 6 | trace | 0.2 | neg | neg |
| H-315 | 470 | neg | neg | neg | 1.015 | neg | 7.5 | neg | 0.2 | neg | neg |
| GMU68 | 470 | neg | neg | neg | 1.015 | neg | 7.5 | neg | 0.2 | neg | neg |
| GMU69 | 470 | neg | neg | neg | 1.015 | neg | 7.5 | neg | 0.2 | neg | neg |
| R-DM007 | 471 | neg | neg | neg | 1.01 | neg | 8.5 | neg | 0.2 | neg | neg |
| R-DM009 | 471 | neg | neg | neg | 1.01 | neg | 8.5 | neg | 0.2 | neg | neg |
| GMU72 | 471 | neg | neg | neg | 1.01 | neg | 8.5 | neg | 0.2 | neg | neg |
| GMU73 | 472 | neg | neg | neg | 1.01 | neg | 7.5 | neg | 0.2 | neg | neg |
| GMU74 | 473 | neg | neg | neg | 1.01 | neg | 8 | neg | 0.2 | neg | neg |
| GMU75 | 473 | neg | neg | neg | 1.01 | neg | 8 | neg | 0.2 | neg | neg |
| GMU76 | 473 | neg | neg | neg | 1.01 | neg | 8 | neg | 0.2 | neg | neg |
| GMU77 | 474 | neg | neg | neg | 1.015 | neg | 6 | neg | 0.2 | neg | neg |
| GMU78 | 474 | neg | neg | neg | 1.015 | neg | 6 | neg | 0.2 | neg | neg |
| GMU79 | 474 | neg | neg | neg | 1.015 | neg | 6 | neg | 0.2 | neg | neg |
| GMU27 | 475 | neg | neg | neg | 1.005 | neg | 6 | neg | 0.2 | neg | neg |
| GMU28 | 475 | neg | neg | neg | 1.005 | neg | 6 | neg | 0.2 | neg | neg |
| L10 | 476 | neg | neg | neg | 1.015 | neg | 6 | neg | 0.2 | neg | neg |
| L11 | 477 | neg | neg | neg | 1.015 | neg | 6 | neg | 0.2 | neg | neg |
| L12 | 477 | neg | neg | neg | 1.015 | neg | 6 | neg | 0.2 | neg | neg |
| L13 | 477 | neg | neg | neg | 1.015 | neg | 6 | neg | 0.2 | neg | neg |
| L14 | 478 | neg | neg | neg | 1.01 | neg | 8 | neg | 0.2 | neg | neg |
| L15 | 479 | neg | neg | neg | 1.01 | neg | 7.5 | neg | 0.2 | neg | neg |
| L16 | 480 | neg | neg | neg | 1.02 | neg | 6 | neg | 0.2 | neg | neg |
| L17 | 481 | neg | neg | neg | 1.01 | trace | 8 | neg | 0.2 | neg | neg |
| L18 | 481 | neg | neg | neg | 1.01 | trace | 8 | neg | 0.2 | neg | neg |
| L19 | 481 | neg | neg | neg | 1.01 | trace | 8 | neg | 0.2 | neg | neg |
| L20 | 482 | neg | neg | neg | 1.02 | neg | 6 | trace | 0.2 | neg | neg |
| L21 | 482 | neg | neg | neg | 1.02 | neg | 6 | trace | 0.2 | neg | neg |
| L22 | 482 | neg | neg | neg | 1.02 | neg | 6 | trace | 0.2 | neg | neg |
| L23 | 483 | neg | neg | neg | 1.01 | neg | 8 | neg | 0.2 | neg | neg |
| L24 | 483 | neg | neg | neg | 1.01 | neg | 8 | neg | 0.2 | neg | neg |
| L25 | 483 | neg | neg | neg | 1.01 | neg | 8 | neg | 0.2 | neg | neg |
| L26 | 484 | neg | neg | neg | 1.015 | neg | 7 | neg | 0.2 | neg | neg |
| L27 | 484 | neg | neg | neg | 1.015 | neg | 7 | neg | 0.2 | neg | neg |
| L28 | 484 | neg | neg | neg | 1.015 | neg | 7 | neg | 0.2 | neg | neg |
| L29 | 485 | neg | neg | neg | 1.01 | neg | 8.5 | neg | 0.2 | neg | neg |
| L30 | 485 | neg | neg | neg | 1.01 | neg | 8.5 | neg | 0.2 | neg | neg |
| L31 | 485 | neg | neg | neg | 1.01 | neg | 8.5 | neg | 0.2 | neg | neg |
| L32 | 486 | neg | neg | neg | 1.015 | neg | 6 | neg | 0.2 | neg | neg |
| L33 | 486 | neg | neg | neg | 1.015 | neg | 6 | neg | 0.2 | neg | neg |
| L34 | 486 | neg | neg | neg | 1.015 | neg | 6 | neg | 0.2 | neg | neg |
| L36 | 487 | neg | neg | neg | 1.015 | neg | 6 | neg | 0.2 | neg | neg |
| L37 | 488 | neg | neg | neg | 1.02 | neg | 6 | trace | 0.2 | neg | neg |
| L38 | 488 | neg | neg | neg | 1.02 | neg | 6 | trace | 0.2 | neg | neg |
| L39 | 488 | neg | neg | neg | 1.02 | neg | 6 | trace | 0.2 | neg | neg |
| L40 | 489 | neg | small | moderate | 1.01 | neg | 8.5 | trace | 0.2 | neg | neg |
| L41 | 489 | neg | small | moderate | 1.01 | neg | 8.5 | trace | 0.2 | neg | neg |
| L42 | 489 | neg | small | moderate | 1.01 | neg | 8.5 | trace | 0.2 | neg | neg |
| L43 | 490 | neg | neg | neg | 1.015 | moderate | 8.5 | trace | 0.2 | neg | trace |
| L44 | 490 | neg | neg | neg | 1.015 | moderate | 8.5 | trace | 0.2 | neg | trace |
| L45 | 490 | neg | neg | neg | 1.015 | moderate | 8.5 | trace | 0.2 | neg | trace |
| L46 | 491 | neg | neg | neg | 1.01 | neg | 6.5 | neg | 0.2 | neg | neg |
| L47 | 491 | neg | neg | neg | 1.01 | neg | 6.5 | neg | 0.2 | neg | neg |
| L48 | 491 | neg | neg | neg | 1.01 | neg | 6.5 | neg | 0.2 | neg | neg |
| L49 | 492 | neg | neg | neg | 1.015 | neg | 7.5 | neg | 0.2 | neg | neg |
| L50 | 492 | neg | neg | neg | 1.015 | neg | 7.5 | neg | 0.2 | neg | neg |
| L51 | 492 | neg | neg | neg | 1.015 | neg | 7.5 | neg | 0.2 | neg | neg |
| L52 | 493 | neg | neg | neg | 1.01 | neg | 6 | neg | 0.2 | neg | neg |
| L53 | 493 | neg | neg | neg | 1.01 | neg | 6 | neg | 0.2 | neg | neg |
| L54 | 493 | neg | neg | neg | 1.01 | neg | 6 | neg | 0.2 | neg | neg |
| L55 | 494 | neg | neg | neg | 1.01 | neg | 8 | neg | 0.2 | neg | neg |
| L56 | 495 | neg | neg | neg | 1.015 | neg | 6.5 | trace | 0.2 | neg | neg |
| L57 | 495 | neg | neg | neg | 1.015 | neg | 6.5 | trace | 0.2 | neg | neg |
| L58 | 495 | neg | neg | neg | 1.015 | neg | 6.5 | trace | 0.2 | neg | neg |
| L59 | 496 | neg | neg | neg | 1.01 | neg | 6.5 | neg | 0.2 | neg | moderate |
| L60 | 496 | neg | neg | neg | 1.01 | neg | 6.5 | neg | 0.2 | neg | moderate |
| L61 | 496 | neg | neg | neg | 1.01 | neg | 6.5 | neg | 0.2 | neg | moderate |
| L63 | 497 | neg | neg | neg | 1.015 | neg | 6 | neg | 0.2 | neg | neg |
| L64 | 497 | neg | neg | neg | 1.015 | neg | 6 | neg | 0.2 | neg | neg |
| L65 | 497 | neg | neg | neg | 1.015 | neg | 6 | neg | 0.2 | neg | neg |
| UW-515 | 498 | neg | neg | neg | 1.01 | trace | 8 | neg | 0.2 | neg | neg |
| L67 | 498 | neg | neg | neg | 1.01 | trace | 8 | neg | 0.2 | neg | neg |
| L68 | 498 | neg | neg | neg | 1.01 | trace | 8 | neg | 0.2 | neg | neg |
| UW-687 | 499 | neg | neg | neg | 1.01 | neg | 8 | neg | 0.2 | neg | neg |
| L70 | 499 | neg | neg | neg | 1.01 | neg | 8 | neg | 0.2 | neg | neg |
| L71 | 499 | neg | neg | neg | 1.01 | neg | 8 | neg | 0.2 | neg | neg |
| UW-500 | 500 | neg | neg | neg | 1.01 | neg | 7.5 | neg | 0.2 | neg | neg |
| L73 | 500 | neg | neg | neg | 1.01 | neg | 7.5 | neg | 0.2 | neg | neg |
| L74 | 501 | neg | neg | neg | 1.02 | neg | 6 | neg | 0.2 | neg | neg |
| L75 | 501 | neg | neg | neg | 1.02 | neg | 6 | neg | 0.2 | neg | neg |
| L76 | 501 | neg | neg | neg | 1.02 | neg | 6 | neg | 0.2 | neg | neg |
| L77 | 502 | neg | small | neg | 1.02 | neg | 6 | trace | 0.2 | neg | neg |
| L78 | 503 | neg | neg | large | 1.02 | neg | 6.5 | neg | 0.2 | neg | neg |
| L79 | 504 | neg | neg | neg | 1.02 | neg | 6 | neg | 0.2 | neg | neg |
| L80 | 505 | neg | neg | neg | 1.01 | neg | 8 | neg | 0.2 | neg | neg |
| L81 | 505 | neg | neg | neg | 1.01 | neg | 8 | neg | 0.2 | neg | neg |
| L82 | 505 | neg | neg | neg | 1.01 | neg | 8 | neg | 0.2 | neg | neg |
| L83 | 506 | neg | neg | neg | 1.015 | neg | 6 | neg | 0.2 | neg | neg |
| L84 | 506 | neg | neg | neg | 1.015 | neg | 6 | neg | 0.2 | neg | neg |
| L85 | 506 | neg | neg | neg | 1.015 | neg | 6 | neg | 0.2 | neg | neg |
| L86 | 507 | neg | neg | neg | 1.005 | neg | 7 | neg | 0.2 | neg | neg |
| L87 | 507 | neg | neg | neg | 1.005 | neg | 7 | neg | 0.2 | neg | neg |
| L88 | 507 | neg | neg | neg | 1.005 | neg | 7 | neg | 0.2 | neg | neg |
| UW-539 | 508 | neg | neg | neg | 1.01 | neg | 8.5 | trace | 0.2 | neg | neg |
| UW-907 | 508 | neg | neg | neg | 1.01 | neg | 8.5 | trace | 0.2 | neg | neg |
| UW-732 | 508 | neg | neg | neg | 1.01 | neg | 8.5 | trace | 0.2 | neg | neg |
| UW-730 | 509 | neg | neg | neg | 1.01 | neg | 7.5 | neg | 0.2 | neg | neg |
| L93 | 510 | neg | neg | trace | 1.005 | neg | 6 | neg | 0.2 | neg | neg |
| L94 | 511 | neg | neg | neg | 1.02 | trace | 6 | trace | 0.2 | neg | neg |
| L95 | 512 | neg | neg | neg | 1.01 | neg | 7.5 | neg | 0.2 | neg | neg |
| UW-535 | 512 | neg | neg | neg | 1.01 | neg | 7.5 | neg | 0.2 | neg | neg |
| L97 | 512 | neg | neg | neg | 1.01 | neg | 7.5 | neg | 0.2 | neg | neg |
| L100 | 513 | neg | neg | neg | 1.01 | neg | 7.5 | neg | 0.2 | neg | neg |
| UW-509 | 513 | neg | neg | neg | 1.01 | neg | 7.5 | neg | 0.2 | neg | neg |
| L99 | 513 | neg | neg | neg | 1.01 | neg | 7.5 | neg | 0.2 | neg | neg |
| L101 | 514 | neg | neg | neg | 1.02 | neg | 6 | neg | 0.2 | neg | trace |
| L102 | 515 | neg | neg | neg | 1.01 | neg | 8.5 | neg | 0.2 | neg | neg |
| L103 | 515 | neg | neg | neg | 1.01 | neg | 8.5 | neg | 0.2 | neg | neg |
| L104 | 515 | neg | neg | neg | 1.01 | neg | 8.5 | neg | 0.2 | neg | neg |
| UW-534 | 516 | neg | neg | neg | 1.015 | neg | 6 | neg | 0.2 | neg | neg |
| GMU100 | 517 | neg | neg | neg | 1.015 | neg | 7 | trace | 0.2 | neg | trace |
| GMU98 | 517 | neg | neg | neg | 1.015 | neg | 7 | trace | 0.2 | neg | trace |
| GMU99 | 517 | neg | neg | neg | 1.015 | neg | 7 | trace | 0.2 | neg | trace |
| GMU101 | 518 | neg | neg | neg | 1.01 | neg | 8.5 | neg | 0.2 | neg | neg |
| UW-647 | 519 | neg | neg | neg | 1.01 | trace | 6.5 | trace | 0.2 | pos | neg |
| GMU103 | 519 | neg | neg | neg | 1.01 | trace | 6.5 | trace | 0.2 | pos | neg |
| GMU104 | 519 | neg | neg | neg | 1.01 | trace | 6.5 | trace | 0.2 | pos | neg |
| GMU105 | 520 | neg | neg | neg | 1.015 | neg | 6 | neg | 0.2 | neg | neg |
| GMU106 | 521 | neg | neg | neg | 1.015 | trace | 6 | trace | 0.2 | pos | moderate |
| L1 | 522 | neg | neg | neg | 1.01 | neg | 7.5 | neg | 0.2 | neg | neg |
| L2 | 523 | neg | neg | neg | 1.005 | neg | 8 | neg | 0.2 | neg | neg |
| L3 | 523 | neg | neg | neg | 1.005 | neg | 8 | neg | 0.2 | neg | neg |
| L4 | 523 | neg | neg | neg | 1.005 | neg | 8 | neg | 0.2 | neg | neg |
| L5 | 523 | neg | neg | neg | 1.005 | neg | 8 | neg | 0.2 | neg | neg |
| L6 | 524 | neg | neg | neg | 1.005 | neg | 8.5 | neg | 0.2 | neg | neg |
| UW-772 | 525 | neg | neg | neg | 1.02 | neg | 6 | trace | 0.2 | neg | neg |
| UW-842 | 526 | neg | neg | large | 1.02 | neg | 6 | neg | 0.2 | neg | neg |
| UW-865 | 527 | neg | small | trace | 1.01 | neg | 6 | pos 1+ | 0.2 | neg | neg |
| UW-867 | 528 | neg | neg | neg | 1.005 | neg | 6 | neg | 0.2 | neg | neg |
| UW-868 | 529 | neg | small | neg | 1.01 | small | 6 | trace | 1 | neg | neg |
| UW-869 | 530 | neg | neg | neg | 1.01 | neg | 8.5 | neg | 0.2 | neg | neg |
| UW-870 | 531 | neg | neg | neg | 1.01 | neg | 7.5 | trace | 0.2 | neg | neg |
| UW-882 | 532 | neg | neg | neg | 1.01 | neg | 7 | trace | 0.2 | neg | neg |
| UW-889 | 533 | neg | neg | neg | 1.01 | neg | 7 | trace | 0.2 | neg | neg |
| UW-890 | 534 | neg | neg | neg | 1.02 | neg | 6.5 | trace | 0.2 | neg | neg |
| UW-891 | 535 | neg | moderate | moderate | 1.015 | neg | 6.5 | pos 1+ | 0.2 | neg | neg |
| UW-894 | 536 | neg | neg | neg | 1.01 | neg | 7.5 | neg | 0.2 | neg | neg |
| UW-898 | 537 | pos 4+ | neg | neg | 1.01 | neg | 6 | trace | 0.2 | neg | moderate |
| UW-900 | 538 | neg | neg | neg | 1.01 | neg | 6 | neg | 0.2 | neg | neg |
| UW-902 | 539 | neg | neg | neg | 1.005 | neg | 6 | neg | 0.2 | neg | neg |
| UW-905 | 540 | neg | neg | neg | 1.01 | neg | 8 | neg | 0.2 | neg | neg |
| UW-906 | 541 | neg | neg | neg | 1.01 | neg | 7 | neg | 0.2 | neg | neg |
| UW-914 | 542 | neg | small | trace | 1.01 | neg | 6 | trace | 0.2 | neg | neg |
| UW-916 | 543 | neg | neg | neg | 1.01 | neg | 6 | neg | 0.2 | neg | neg |

**Supplementary Table 3**. Values of urinary LAM concentration in TB patients and controls (N=430) measured using MoAb1, CS35, and A194 antibodies.

| **ID #** | **TB** | **HIV** | **MoAb1** | **CS35** | **A194** | **Country_cases** |
| --- | --- | --- | --- | --- | --- | --- |
| O-DM001 | 1 | 0 | 0.264902 | 0.089096 | 0.028638 | 1 |
| O-DM002 | 1 | 0 | 0.049321 | 0.023865 | 0.04773 | 1 |
| O-DM003 | 1 | 1 | 0.039775 | 1.186091 | 0.092278 | 1 |
| O-DM004 | 1 | 0 | 0.109779 | 0.018297 | 0.122507 | 1 |
| O-DM005 | 1 | 1 | 3.767488 | 26.66118 | 0.713564 | 1 |
| O-DM006 | 1 | 0 | 1.172567 | 1.354737 | 0.157509 | 1 |
| O-DM008 | 1 | 0 | 0.00462 | 5.986138 | 0.774022 | 1 |
| O-DM009 | 1 | 0 | 0.004616 | 0.97051 | 0.311836 | 1 |
| O-DM010 | 1 | 0 | 0.004616 | 2.209899 | 0.182965 | 1 |
| O-DM011 | 1 | 0 | 0.978465 | 0.06364 | 0.007955 | 1 |
| O-DM012 | 1 | 0 | 1.680892 | 0.248992 | 0.233877 | 1 |
| O-DM013 | 1 | 0 | 0.029434 | 0.187738 | 0.151941 | 1 |
| O-DM014 | 1 | 0 | 0.050912 | 0.040571 | 0.152736 | 1 |
| O-DM014 | 1 | 0 | 0.081937 | 0.515484 | 0.264902 | 1 |
| O-DM015 | 1 | 0 | 1.32053 | 0.04773 | 0.532985 | 1 |
| O-DM016 | 1 | 0 | 0.803455 | 0.109779 | 0.136826 | 1 |
| O-DM017 | 1 | 0 | 2.521735 | 0.00433 | 0.119325 | 1 |
| O-DM018 | 1 | 0 | 0.81141 | 0.01591 | 0.055685 | 1 |
| O-DM019 | 1 | 0 | 0.707995 | 0.141599 | 0.009546 | 1 |
| O-DM020 | 1 | 0 | 4.558215 | 0.00433 | 0.105006 | 1 |
| O-DM021 | 1 | 0 | 0.42957 | 0.058072 | 0.041366 | 1 |
| O-DM022 | 1 | 0 | 1.423945 | 0.259333 | 0.009546 | 1 |
| O-DM023 | 1 | 0 | 1.567135 | 0.00433 | 0.105006 | 1 |
| O-DM024 | 1 | 0 | 3.85022 | 0.048526 | 0.075573 | 1 |
| O-DM025 | 1 | 0 | 1.233025 | 1.23223 | 0.026252 | 1 |
| O-DM026 | 1 | 0 | 0.90687 | 0.173419 | 0.265697 | 1 |
| O-DM027 | 1 | 1 | 0.081141 | 1.554407 | 0.251378 | 1 |
| O-DM028 | 1 | 0 | 0.217172 | 0.078755 | 0.094665 | 1 |
| O-DM029 | 1 | 0 | 0.004773 | 0.85914 | 0.326155 | 1 |
| O-DM030 | 1 | 0 | 3.232117 | 0.389795 | 1.423945 | 1 |
| O-DM031 | 1 | 0 | 0.269675 | 0.215581 | 0.241832 | 1 |
| O-DM032 | 1 | 0 | 0.004616 | 0.077164 | 0.149554 | 1 |
| O-DM033 | 1 | 0 | 0.809024 | 0.077164 | 0.082732 | 1 |
| O-HC501 | 1 | 0 | 0.104211 | 0.216376 | 0.130462 | 1 |
| O-HC502 | 1 | 0 | 0.004616 | 0.49321 | 0.178192 | 1 |
| O-HC503 | 1 | 0 | 1.532929 | 0.112166 | 0.202057 | 1 |
| O-HC504 | 1 | 1 | 2.2274 | 0.365135 | 0.295131 | 1 |
| O-HC506 | 1 | 0 | 0.14319 | 0.004328 | 0.03182 | 1 |
| O-HC507 | 1 | 0 | 0.73902 | 0.33411 | 0.06364 | 1 |
| O-HC509 | 1 | 0 | 0.408092 | 0.326155 | 0.22274 | 1 |
| O-HC510 | 1 | 1 | 0.016706 | 1.344395 | 0.004495 | 1 |
| O-HC511 | 1 | 0 | 0.62049 | 0.00433 | 0.071595 | 1 |
| O-HC512 | 1 | 0 | 0.00462 | 0.055685 | 0.023865 | 1 |
| O-HC513 | 1 | 0 | 0.622877 | 0.017501 | 0.100233 | 1 |
| O-HC514 | 1 | 0 | 2.8638 | 0.004328 | 0.004495 | 1 |
| O-HC514 | 1 | 0 | 0.106597 | 0.004328 | 0.004495 | 1 |
| O-HC515 | 1 | 0 | 0.00462 | 0.00433 | 0.004495 | 1 |
| O-HC516 | 1 | 0 | 0.106597 | 0.039775 | 0.03182 | 1 |
| O-HC517 | 1 | 0 | 0.57276 | 0.06364 | 0.004495 | 1 |
| O-HC518 | 1 | 0 | 0.529803 | 1.63873 | 0.103415 | 1 |
| O-HC520 | 1 | 0 | 0.30229 | 4.255925 | 0.20683 | 1 |
| O-HC521 | 1 | 0 | 5.518384 | 5.32985 | 0.20683 | 1 |
| O-HC522 | 1 | 0 | 0.406501 | 4.017275 | 0.182965 | 1 |
| O-HC523 | 1 | 0 | 0.00462 | 0.042162 | 0.105006 | 1 |
| O-HC524 | 1 | 0 | 3.660096 | 1.977613 | 0.487642 | 1 |
| O-HC525 | 1 | 0 | 1.772374 | 0.434343 | 0.459004 | 1 |
| O-HC526 | 1 | 0 | 3.963977 | 3.091313 | 1.633162 | 1 |
| O-HC528Â Â | 1 | 0 | 0.113757 | 0.612535 | 0.03182 | 1 |
| O-HC529 | 1 | 0 | 3.287006 | 3.837492 | 0.470141 | 1 |
| O-HC530 | 1 | 0 | 2.400024 | 1.449401 | 0.370703 | 1 |
| O-HC531 | 1 | 0 | 0.004616 | 0.039775 | 0.039775 | 1 |
| O-HC532 | 1 | 0 | 0.062049 | 0.210808 | 0.040571 | 1 |
| O-HC533 | 1 | 0 | 0.162282 | 0.393773 | 0.155918 | 1 |
| O-HC534 | 1 | 0 | 2.68879 | 4.257516 | 1.154271 | 1 |
| O-HC535 | 1 | 0 | 1.089835 | 0.389795 | 0.103415 | 1 |
| O-HC537 | 1 | 0 | 0.034207 | 0.185352 | 0.131258 | 1 |
| O-HC538 | 1 | 0 | 0.58867 | 0.60458 | 0.564805 | 1 |
| O-HC539 | 1 | 0 | 0.00462 | 0.00433 | 0.007955 | 1 |
| O-HC540 | 1 | 0 | 0.92278 | 0.87505 | 0.501165 | 1 |
| O-HC541 | 1 | 0 | 0.088301 | 0.240241 | 0.087505 | 1 |
| O-HC542 | 1 | 1 | 0.170237 | 0.925962 | 0.169442 | 1 |
| R-DM013 | 1 | 1 | 1.010285 | 0.262515 | 0.1591 | 1 |
| R-DM020 | 1 | 1 | 0.136031 | 0.46139 | 0.229104 | 1 |
| R-DM022 | 1 | 1 | 0.004616 | 1.011876 | 0.045344 | 1 |
| R-DM024 | 1 | 1 | 0.03182 | 0.052503 | 0.338883 | 1 |
| R-SC008 | 1 | 1 | 0.03182 | 0.01591 | 0.469345 | 1 |
| R-SC018 | 1 | 1 | 0.1591 | 0.4773 | 0.007955 | 1 |
| R-TC002 | 1 | 1 | 0.119325 | 0.023865 | 0.39775 | 1 |
| O-HC505 | 1 | 0 | 0.260129 | 0.033411 | 0.021479 | 1 |
| O-HC536 | 1 | 0 | 4.364909 | 2.505825 | 2.193194 | 1 |
| GB-10A | 1 | 0 | 0.214785 | 0.00433 | 0.004495 | 2 |
| GB-13A | 1 | 1 | 0.54094 | 0.00433 | 0.071595 | 2 |
| GB-14A | 1 | 1 | 2.362635 | 0.00433 | 0.004495 | 2 |
| GB-15A | 1 | 0 | 0.006364 | 0.230695 | 0.004495 | 2 |
| GB-16A | 1 | 0 | 0.474118 | 0.57276 | 0.03182 | 2 |
| GB-17A | 1 | 1 | 0.65231 | 0.248992 | 0.050912 | 2 |
| GB-18A | 1 | 1 | 1.899654 | 0.231491 | 0.251378 | 2 |
| GB-1A | 1 | 1 | 0.835275 | 0.00433 | 0.06364 | 2 |
| GB-3A | 1 | 1 | 0.46139 | 0.00433 | 0.03182 | 2 |
| GB-4A | 1 | 0 | 0.004616 | 0.962555 | 0.004495 | 2 |
| GB-5A | 1 | 0 | 1.46372 | 0.00433 | 0.01591 | 2 |
| GB-6A | 1 | 0 | 7.947045 | 0.00433 | 0.007955 | 2 |
| GB-7A | 1 | 0 | 5.226435 | 0.03182 | 0.30229 | 2 |
| GB-8A | 1 | 0 | 3.921815 | 0.00433 | 0.071595 | 2 |
| GB-12A | 1 | 0 | 0.402523 | 0.373885 | 0.055685 | 2 |
| GB-2A | 1 | 0 | 0.09546 | 0.00433 | 0.004495 | 2 |
| GB-9A | 1 | 0 | 0.268084 | 0.00433 | 0.03182 | 2 |
| U-NTT155 | 1 | 0 | 0.09546 | 0.103415 | 0.1591 | 3 |
| U-NTT162 | 1 | 0 | 1.4319 | 1.478835 | 0.464572 | 3 |
| U-NTT173 | 1 | 0 | 1.4319 | 0.395364 | 0.377067 | 3 |
| U-NTT179 | 1 | 0 | 1.869425 | 3.969545 | 1.38417 | 3 |
| U-NTT181 | 1 | 0 | 0.596625 | 0.089096 | 0.085119 | 3 |
| U-NTT192 | 1 | 0 | 0.49321 | 0.004328 | 0.04773 | 3 |
| U-NTT197 | 1 | 0 | 1.35235 | 1.042105 | 0.58867 | 3 |
| UW-117 | 1 | 1 | 0.502756 | 1.76601 | 0.585488 | 3 |
| UW-119 | 1 | 0 | 0.25456 | 1.551225 | 0.197284 | 3 |
| UW-120 | 1 | 0 | 0.042957 | 0.004328 | 0.425593 | 3 |
| UW-122 | 1 | 1 | 0.091483 | 2.43423 | 0.200466 | 3 |
| UW-124 | 1 | 0 | 0.560828 | 1.758055 | 0.173419 | 3 |
| UW-476 | 1 | 0 | 0.005569 | 0.004328 | 0.091483 | 3 |
| UW-477 | 1 | 1 | 0.210808 | 0.11137 | 0.214785 | 3 |
| UW-482 | 1 | 1 | 2.002274 | 3.61157 | 0.637196 | 3 |
| UW-485 | 1 | 1 | 0.138417 | 7.242232 | 0.284789 | 3 |
| UW-491 | 1 | 1 | 0.004616 | 0.05489 | 0.075573 | 3 |
| UW-501 | 1 | 1 | 0.14319 | 0.485255 | 0.58867 | 3 |
| UW-502 | 1 | 0 | 0.07955 | 0.20683 | 0.004495 | 3 |
| UW-504 | 1 | 0 | 0.946645 | 1.598955 | 0.39775 | 3 |
| UW-505 | 1 | 0 | 0.007955 | 0.023865 | 0.03182 | 3 |
| UW-507 | 1 | 1 | 0.819365 | 0.98642 | 0.11137 | 3 |
| UW-531 | 1 | 1 | 0.421615 | 0.46139 | 0.182965 | 3 |
| UW-578 | 1 | 1 | 1.78192 | 2.45014 | 0.946645 | 3 |
| UW-611 | 1 | 1 | 1.455765 | 2.012615 | 0.3182 | 3 |
| UW-649 | 1 | 1 | 4.50253 | 0.81141 | 0.33411 | 3 |
| UW-656 | 1 | 1 | 1.12961 | 5.90261 | 0.596625 | 3 |
| UW-667 | 1 | 1 | 0.03182 | 0.692085 | 0.135235 | 3 |
| UW-668 | 1 | 1 | 4.08887 | 3.83431 | 0.278425 | 3 |
| UW-685 | 1 | 1 | 0.230695 | 3.69112 | 0.42957 | 3 |
| UW-706 | 1 | 1 | 0.12728 | 0.3182 | 0.326155 | 3 |
| UW-707 | 1 | 1 | 0.00462 | 0.60458 | 0.82732 | 3 |
| UW-718 | 1 | 1 | 1.360305 | 3.635435 | 0.93869 | 3 |
| UW-721 | 1 | 1 | 0.28638 | 0.835275 | 0.437525 | 3 |
| UW-722 | 1 | 1 | 1.25689 | 0.85914 | 1.805785 | 3 |
| UW-733 | 1 | 1 | 0.46139 | 1.51145 | 0.36593 | 3 |
| UW-735 | 1 | 1 | 2.314905 | 5.656005 | 2.00466 | 3 |
| UW-736 | 1 | 0 | 0.861527 | 5.7276 | 0.478891 | 3 |
| UW-738 | 1 | 0 | 0.010342 | 0.004328 | 0.027047 | 3 |
| UW-739 | 1 | 0 | 0.004616 | 0.004328 | 0.030229 | 3 |
| UW-740 | 1 | 0 | 1.909996 | 1.19325 | 0.949032 | 3 |
| UW-741 | 1 | 0 | 0.030229 | 0.871868 | 0.136031 | 3 |
| UW-742 | 1 | 1 | 2.24331 | 3.38883 | 0.596625 | 3 |
| UW-743 | 1 | 0 | 0.258538 | 0.026252 | 0.283198 | 3 |
| UW-744 | 1 | 0 | 0.11853 | 0.81141 | 0.272061 | 3 |
| UW-745 | 1 | 0 | 2.599694 | 8.784707 | 0.726292 | 3 |
| UW-757 | 1 | 1 | 0.087505 | 4.1366 | 0.25456 | 3 |
| UW-758 | 1 | 0 | 0.52503 | 0.004328 | 0.25456 | 3 |
| UW-763 | 1 | 0 | 1.00233 | 0.3182 | 0.262515 | 3 |
| UW-764 | 1 | 1 | 0.50912 | 1.726235 | 0.373885 | 3 |
| UW-766 | 1 | 0 | 1.20916 | 1.60691 | 0.58867 | 3 |
| UW-767 | 1 | 0 | 0.771635 | 0.851185 | 0.262515 | 3 |
| UW-768 | 1 | 0 | 0.326155 | 0.68413 | 0.310245 | 3 |
| UW-771 | 1 | 0 | 1.47963 | 1.455765 | 0.278425 | 3 |
| UW-772 | 1 | 0 | 0.007955 | 0.01591 | 0.007955 | 3 |
| UW-774 | 1 | 0 | 0.230695 | 0.58867 | 0.198875 | 3 |
| UW-775 | 1 | 1 | 0.517075 | 0.46139 | 0.19092 | 3 |
| UW-783 | 1 | 0 | 0.68413 | 0.564805 | 0.19092 | 3 |
| UW-784 | 1 | 0 | 0.389795 | 0.437525 | 0.214785 | 3 |
| UW-788 | 1 | 0 | 0.49321 | 0.342065 | 0.135235 | 3 |
| UW-793 | 1 | 1 | 0.405705 | 1.328485 | 0.421615 | 3 |
| UW-794 | 1 | 0 | 0.182965 | 0.230695 | 0.23865 | 3 |
| UW-799 | 1 | 0 | 0.119325 | 0.36593 | 0.11137 | 3 |
| UW-803 | 1 | 0 | 7.77999 | 5.926475 | 1.121655 | 3 |
| UW-805 | 1 | 1 | 2.283085 | 6.639243 | 1.24098 | 3 |
| UW-807 | 1 | 0 | 7.65271 | 2.95926 | 0.135235 | 3 |
| UW-808 | 1 | 1 | 5.93443 | 5.48895 | 0.39775 | 3 |
| UW-811 | 1 | 1 | 0.214785 | 2.45014 | 0.182965 | 3 |
| UW-812 | 1 | 0 | 0.09546 | 0.039775 | 0.198875 | 3 |
| UW-814 | 1 | 0 | 0.580715 | 1.980795 | 0.310245 | 3 |
| UW-817 | 1 | 1 | 0.103415 | 3.56384 | 0.151145 | 3 |
| UW-818 | 1 | 0 | 2.27513 | 3.05472 | 0.9546 | 3 |
| UW-820 | 1 | 0 | 0.01591 | 0.7955 | 0.437525 | 3 |
| UW-823 | 1 | 0 | 0.71595 | 1.60691 | 0.151145 | 3 |
| UW-831 | 1 | 0 | 3.57975 | 4.15251 | 2.24331 | 3 |
| UW-842 | 1 | 0 | 0.167055 | 0.469345 | 0.246605 | 3 |
| UW-845 | 1 | 1 | 1.70237 | 23.2286 | 2.076255 | 3 |
| UW-851 | 1 | 1 | 3.72294 | 22.21832 | 0.55685 | 3 |
| UW-865 | 1 | 0 | 2.617195 | 1.948975 | 1.67055 | 3 |
| UW-867 | 1 | 0 | 0.373885 | 0.25456 | 0.17501 | 3 |
| UW-868 | 1 | 0 | 1.9092 | 2.11603 | 0.914825 | 3 |
| UW-869 | 1 | 0 | 0.007955 | 0.004328 | 0.039775 | 3 |
| UW-870 | 1 | 0 | 0.20683 | 0.07955 | 0.087505 | 3 |
| UW-871 | 1 | 1 | 0.04773 | 4.70936 | 0.294335 | 3 |
| UW-876 | 1 | 1 | 3.460425 | 12.22684 | 0.485255 | 3 |
| UW-882 | 1 | 0 | 0.182965 | 0.12728 | 0.20683 | 3 |
| UW-889 | 1 | 0 | 0.38184 | 0.437525 | 0.33411 | 3 |
| UW-890 | 1 | 0 | 0.27047 | 0.453435 | 0.23865 | 3 |
| UW-891 | 1 | 0 | 2.19558 | 1.694415 | 1.08188 | 3 |
| UW-894 | 1 | 0 | 0.007955 | 0.004328 | 0.023865 | 3 |
| UW-898 | 1 | 0 | 0.3182 | 0.294335 | 0.19092 | 3 |
| UW-900 | 1 | 0 | 0.14319 | 0.14319 | 0.135235 | 3 |
| UW-902 | 1 | 0 | 0.12728 | 0.11137 | 0.071595 | 3 |
| UW-905 | 1 | 0 | 0.11137 | 0.055685 | 0.1591 | 3 |
| UW-906 | 1 | 0 | 0.055685 | 0.039775 | 0.12728 | 3 |
| UW-914 | 1 | 0 | 0.89096 | 0.76368 | 0.52503 | 3 |
| UW-916 | 1 | 0 | 2.060345 | 0.198875 | 0.23865 | 3 |
| V-15 | 1 | 0 | 0.14 | 0.801751 | 2.126164 | 4 |
| V-38 | 1 | 0 | 2.232173 | 3.419855 | 2.715837 | 4 |
| V-50 | 1 | 0 | 0.517871 | 0.914825 | 0.154327 | 4 |
| V-51 | 1 | 0 | 2.123985 | 3.378489 | 0.873459 | 4 |
| V-53 | 1 | 0 | 0.004616 | 0.42957 | 0.109779 | 4 |
| V-73 | 1 | 0 | 0.348857 | 0.458907 | 0.670944 | 4 |
| V-28 | 1 | 0 | 0.330133 | 0.338883 | 0.058867 | 4 |
| V-30 | 1 | 0 | 0.163078 | 0.735838 | 0.192511 | 4 |
| V-31 | 1 | 0 | 0.039775 | 0.60458 | 0.09546 | 4 |
| V-33 | 1 | 0 | 1.836014 | 0.319791 | 0.087505 | 4 |
| V-34 | 1 | 0 | 1.64589 | 3.139043 | 0.195693 | 4 |
| V-36 | 1 | 0 | 2.085006 | 1.536111 | 0.249787 | 4 |
| V-39 | 1 | 0 | 5.405423 | 1.633162 | 0.348429 | 4 |
| V-40 | 1 | 0 | 2.356271 | 1.020627 | 0.505938 | 4 |
| V-41 | 1 | 0 | 1.106541 | 0.186147 | 0.036593 | 4 |
| V-44 | 1 | 0 | 0.836866 | 1.489972 | 0.290358 | 4 |
| V-46 | 1 | 0 | 0.280016 | 0.319791 | 0.119325 | 4 |
| V-54 | 1 | 0 | 1.210751 | 0.602194 | 0.078755 | 4 |
| V-55 | 1 | 0 | 2.95926 | 4.51844 | 1.710325 | 4 |
| V-57 | 1 | 0 | 2.076255 | 2.983125 | 0.962555 | 4 |
| V-58 | 1 | 0 | 3.91386 | 1.70237 | 0.258538 | 4 |
| V-59 | 1 | 0 | 1.985568 | 0.085914 | 0.0708 | 4 |
| V-60 | 1 | 0 | 1.592591 | 1.204387 | 0.211603 | 4 |
| V-64 | 1 | 0 | 4.363318 | 1.388148 | 0.30945 | 4 |
| V-65 | 1 | 0 | 0.771635 | 0.93869 | 0.33411 | 4 |
| GMU1 | 0 | 0 | 0.07154 | 0.026621 | 0.004495 | 5 |
| GMU10 | 0 | 0 | 0.085445 | 0.062097 | 0.016912 | 5 |
| GMU100 | 0 | 0 | 0.012912 | 0.023382 | 0.078152 | 5 |
| GMU101 | 0 | 0 | 0.004616 | 0.044124 | 0.012211 | 5 |
| UW-647 | 0 | 0 | 0.004616 | 0.004327 | 0.013209 | 5 |
| GMU103 | 0 | 0 | 0.033366 | 0.011422 | 0.067479 | 5 |
| GMU104 | 0 | 0 | 0.013353 | 0.073202 | 0.039077 | 5 |
| GMU105 | 0 | 0 | 0.004616 | 0.061892 | 0.010953 | 5 |
| GMU106 | 0 | 0 | 0.004616 | 0.55455 | 0.016085 | 5 |
| UW-542 | 0 | 0 | 0.004615 | 0.00432 | 0.170000 | 5 |
| GMU12 | 0 | 0 | 0.081622 | 0.057767 | 0.009532 | 5 |
| GMU13 | 0 | 0 | 0.110958 | 0.028412 | 0.035906 | 5 |
| GMU14 | 0 | 0 | 0.078411 | 0.026621 | 0.09439 | 5 |
| UW-533 | 0 | 0 | 0.004615 | 0.560000 | 0.98000 | 5 |
| GMU16 | 0 | 0 | 0.018649 | 0.004328 | 0.004495 | 5 |
| GMU17 | 0 | 0 | 0.011896 | 0.004328 | 0.004495 | 5 |
| UW-750 | 0 | 0 | 0.050000 | 0.004327 | 0.130000 | 5 |
| GMU19 | 0 | 0 | 0.025732 | 0.109251 | 0.096891 | 5 |
| GMU2 | 0 | 0 | 0.07154 | 0.026621 | 0.015958 | 5 |
| GMU20 | 0 | 0 | 0.013179 | 0.015932 | 0.069032 | 5 |
| GMU21 | 0 | 0 | 0.013592 | 0.004328 | 0.258275 | 5 |
| GMU22 | 0 | 0 | 0.046193 | 0.004328 | 0.037406 | 5 |
| GMU23 | 0 | 0 | 0.044362 | 0.004328 | 0.004495 | 5 |
| GMU24 | 0 | 0 | 0.094294 | 0.214692 | 0.078291 | 5 |
| UW-543 | 0 | 0 | 0.004615 | 0.580000 | 0.030000 | 5 |
| UW-500 | 0 | 0 | 0.080000 | 0.004327 | 0.280000 | 5 |
| GMU27 | 0 | 0 | 0.004616 | 0.018081 | 0.012346 | 5 |
| GMU28 | 0 | 0 | 0.011755 | 0.034142 | 0.045523 | 5 |
| GMU29 | 0 | 0 | 0.010645 | 0.046032 | 0.044652 | 5 |
| GMU3 | 0 | 0 | 0.072959 | 0.03048 | 0.004495 | 5 |
| GMU30 | 0 | 0 | 0.004616 | 0.05545 | 0.192908 | 5 |
| GMU31 | 0 | 0 | 0.249655 | 0.27075 | 0.113088 | 5 |
| GMU32 | 0 | 0 | 0.081804 | 0.085252 | 0.038386 | 5 |
| GMU33 | 0 | 0 | 0.011316 | 0.032875 | 0.043701 | 5 |
| GMU34 | 0 | 0 | 0.075374 | 0.029976 | 0.017746 | 5 |
| GMU35 | 0 | 0 | 0.057188 | 0.007334 | 0.040564 | 5 |
| GMU36 | 0 | 0 | 0.010326 | 0.062129 | 0.043701 | 5 |
| GMU37 | 0 | 0 | 0.004616 | 0.035366 | 0.009971 | 5 |
| GMU38 | 0 | 0 | 0.004616 | 0.041459 | 0.010945 | 5 |
| GMU39 | 0 | 0 | 0.004616 | 0.038544 | 0.01064 | 5 |
| GMU4 | 0 | 0 | 0.276646 | 0.03904 | 0.004495 | 5 |
| GMU40 | 0 | 0 | 0.007291 | 0.027368 | 0.043701 | 5 |
| GMU41 | 0 | 0 | 0.007291 | 0.029761 | 0.043701 | 5 |
| GMU42 | 0 | 0 | 0.021308 | 0.030166 | 0.044939 | 5 |
| GMU43 | 0 | 0 | 0.020842 | 0.038216 | 0.049105 | 5 |
| GMU44 | 0 | 0 | 0.007291 | 0.035025 | 0.043701 | 5 |
| GMU45 | 0 | 0 | 0.01294 | 0.033569 | 0.046715 | 5 |
| GMU46 | 0 | 0 | 0.007291 | 0.026621 | 0.045177 | 5 |
| GMU47 | 0 | 0 | 0.007291 | 0.405879 | 0.043701 | 5 |
| GMU48 | 0 | 0 | 0.009717 | 0.064996 | 0.049796 | 5 |
| GMU49 | 0 | 0 | 0.011884 | 0.070184 | 0.043701 | 5 |
| GMU5 | 0 | 0 | 0.073805 | 0.026621 | 0.004495 | 5 |
| GMU50 | 0 | 0 | 0.026419 | 0.342606 | 0.043701 | 5 |
| UW-543 | 0 | 0 | 0.004615 | 0.85000 | 0.030000 | 5 |
| UW-734 | 0 | 0 | 0.004615 | 0.54000 | 0.050000 | 5 |
| GMU53 | 0 | 0 | 0.130015 | 0.059187 | 0.060581 | 5 |
| GMU54 | 0 | 0 | 0.064122 | 0.1621 | 0.048577 | 5 |
| GMU55 | 0 | 0 | 0.224035 | 0.034718 | 0.049227 | 5 |
| GMU56 | 0 | 0 | 1.772297 | 0.172535 | 0.039794 | 5 |
| GMU57 | 0 | 0 | 0.239659 | 0.022198 | 0.072403 | 5 |
| GMU58 | 0 | 0 | 0.110871 | 0.100571 | 0.05445 | 5 |
| GMU59 | 0 | 0 | 0.231565 | 0.196021 | 0.004495 | 5 |
| GMU6 | 0 | 0 | 0.131662 | 0.026621 | 0.004495 | 5 |
| GMU60 | 0 | 0 | 0.08494 | 0.025774 | 0.156031 | 5 |
| GMU61 | 0 | 0 | 0.014571 | 0.039452 | 0.048791 | 5 |
| GMU62 | 0 | 0 | 0.09565 | 0.086012 | 0.044169 | 5 |
| GMU63 | 0 | 0 | 0.065805 | 0.015548 | 0.043676 | 5 |
| GMU64 | 0 | 0 | 0.009062 | 0.031851 | 0.044988 | 5 |
| GMU65 | 0 | 0 | 0.024465 | 0.372704 | 0.004495 | 5 |
| GMU66 | 0 | 0 | 0.018106 | 0.090324 | 0.055224 | 5 |
| GMU67 | 0 | 0 | 0.03213 | 0.08961 | 0.101186 | 5 |
| H-315 | 0 | 0 | 0.220000 | 0.070000 | 0.08000 | 5 |
| GMU69 | 0 | 0 | 0.090642 | 0.013493 | 0.03106 | 5 |
| GMU7 | 0 | 0 | 0.084527 | 0.026621 | 0.004495 | 5 |
| R-DM007 | 0 | 0 | 0.004615 | 0.170000 | 0.010000 | 5 |
| R-DM009 | 0 | 0 | 0.004615 | 0.004327 | 0.100000 | 5 |
| GMU72 | 0 | 0 | 0.071915 | 0.034841 | 0.076921 | 5 |
| GMU73 | 0 | 0 | 0.060028 | 0.028825 | 0.056867 | 5 |
| GMU74 | 0 | 0 | 0.01312 | 0.029979 | 0.043701 | 5 |
| GMU75 | 0 | 0 | 0.07154 | 0.232276 | 0.115521 | 5 |
| GMU76 | 0 | 0 | 0.049936 | 0.011821 | 0.004495 | 5 |
| GMU77 | 0 | 0 | 0.026619 | 0.045555 | 0.048202 | 5 |
| GMU78 | 0 | 0 | 0.135893 | 0.279722 | 0.004495 | 5 |
| GMU79 | 0 | 0 | 0.069926 | 0.013269 | 0.057364 | 5 |
| GMU8 | 0 | 0 | 0.25614 | 0.026621 | 0.004495 | 5 |
| GMU80 | 0 | 0 | 0.004616 | 0.078436 | 0.156788 | 5 |
| GMU81 | 0 | 0 | 0.004616 | 0.004328 | 0.004495 | 5 |
| GMU82 | 0 | 0 | 0.004616 | 0.004328 | 0.004495 | 5 |
| GMU83 | 0 | 0 | 0.004616 | 0.004328 | 0.016019 | 5 |
| GMU84 | 0 | 0 | 0.046725 | 0.005404 | 0.004495 | 5 |
| GMU85 | 0 | 0 | 0.045793 | 0.005404 | 0.004495 | 5 |
| GMU86 | 0 | 0 | 0.049039 | 0.005404 | 0.181488 | 5 |
| GMU87 | 0 | 0 | 0.147889 | 0.005404 | 0.0609 | 5 |
| GMU88 | 0 | 0 | 0.059267 | 0.014332 | 0.008483 | 5 |
| GMU89 | 0 | 0 | 0.073466 | 0.022917 | 0.066427 | 5 |
| GMU9 | 0 | 0 | 0.236325 | 0.709544 | 0.004495 | 5 |
| GMU90 | 0 | 0 | 0.056962 | 0.005404 | 0.01943 | 5 |
| GMU91 | 0 | 0 | 0.101518 | 0.007943 | 0.100177 | 5 |
| GMU92 | 0 | 0 | 0.066534 | 0.015317 | 0.040281 | 5 |
| GMU93 | 0 | 0 | 0.043701 | 0.008053 | 0.119689 | 5 |
| GMU94 | 0 | 0 | 0.055798 | 0.009046 | 0.091156 | 5 |
| GMU95 | 0 | 0 | 0.062072 | 0.005583 | 0.004495 | 5 |
| GMU96 | 0 | 0 | 0.060136 | 0.007123 | 0.004495 | 5 |
| GMU97 | 0 | 0 | 0.053085 | 0.041386 | 0.018745 | 5 |
| GMU98 | 0 | 0 | 0.004616 | 0.050167 | 0.009835 | 5 |
| GMU99 | 0 | 0 | 0.050679 | 0.011422 | 0.017003 | 5 |
| L1 | 0 | 0 | 0.004616 | 0.297462 | 0.011255 | 5 |
| L10 | 0 | 0 | 0.038841 | 0.060003 | 0.010999 | 5 |
| L100 | 0 | 0 | 0.010257 | 0.237743 | 0.224589 | 5 |
| L101 | 0 | 0 | 0.004616 | 1.871919 | 0.017265 | 5 |
| L102 | 0 | 0 | 0.696219 | 0.307298 | 0.028029 | 5 |
| L103 | 0 | 0 | 0.004616 | 0.014211 | 0.119716 | 5 |
| L104 | 0 | 0 | 0.333007 | 0.004328 | 0.134587 | 5 |
| UW-534 | 0 | 0 | 0.004615 | 0.004327 | 0.090000 | 5 |
| L11 | 0 | 0 | 0.004616 | 0.019287 | 0.012165 | 5 |
| L12 | 0 | 0 | 0.07154 | 0.02841 | 0.050679 | 5 |
| L13 | 0 | 0 | 0.061895 | 0.035781 | 0.004495 | 5 |
| L14 | 0 | 0 | 0.004616 | 0.011246 | 0.011451 | 5 |
| L15 | 0 | 0 | 0.004616 | 0.013182 | 0.010984 | 5 |
| L16 | 0 | 0 | 0.004616 | 0.013834 | 0.009835 | 5 |
| L17 | 0 | 0 | 0.48491 | 0.025693 | 0.01909 | 5 |
| L18 | 0 | 0 | 0.498922 | 0.028193 | 0.033366 | 5 |
| L19 | 0 | 0 | 0.049179 | 0.010197 | 0.065881 | 5 |
| L2 | 0 | 0 | 0.01591 | 0.004328 | 0.01591 | 5 |
| L20 | 0 | 0 | 0.004616 | 0.041065 | 0.010541 | 5 |
| L21 | 0 | 0 | 0.076206 | 0.03777 | 0.006313 | 5 |
| L22 | 0 | 0 | 0.043701 | 0.007367 | 0.043245 | 5 |
| L23 | 0 | 0 | 0.396756 | 0.064955 | 0.075918 | 5 |
| L24 | 0 | 0 | 0.004616 | 0.011422 | 0.093091 | 5 |
| L25 | 0 | 0 | 0.015654 | 0.004328 | 0.039834 | 5 |
| L26 | 0 | 0 | 0.004616 | 0.019563 | 0.011802 | 5 |
| L27 | 0 | 0 | 0.015958 | 0.011422 | 0.004495 | 5 |
| L28 | 0 | 0 | 0.009835 | 0.204555 | 0.004495 | 5 |
| L29 | 0 | 0 | 0.004616 | 0.011909 | 0.009945 | 5 |
| L3 | 0 | 0 | 0.004616 | 0.030896 | 0.010806 | 5 |
| L30 | 0 | 0 | 0.004616 | 0.011422 | 0.004495 | 5 |
| L31 | 0 | 0 | 0.009835 | 0.094921 | 0.004495 | 5 |
| L32 | 0 | 0 | 0.004616 | 0.024565 | 0.011224 | 5 |
| L33 | 0 | 0 | 0.004616 | 0.011422 | 0.004495 | 5 |
| L34 | 0 | 0 | 0.010407 | 0.004328 | 0.004495 | 5 |
| L35 | 0 | 0 | 0.004616 | 1.526626 | 0.126761 | 5 |
| L36 | 0 | 0 | 0.134346 | 0.072362 | 0.025949 | 5 |
| L37 | 0 | 0 | 4.71912 | 0.255026 | 0.024461 | 5 |
| L38 | 0 | 0 | 0.004616 | 0.060943 | 0.008896 | 5 |
| L39 | 0 | 0 | 0.146087 | 0.063942 | 0.023396 | 5 |
| L4 | 0 | 0 | 0.006313 | 0.011929 | 0.016766 | 5 |
| L40 | 0 | 0 | 0.238805 | 0.070155 | 0.009835 | 5 |
| L41 | 0 | 0 | 0.004616 | 0.042669 | 0.004495 | 5 |
| L42 | 0 | 0 | 0.028619 | 0.197688 | 0.039237 | 5 |
| L43 | 0 | 0 | 2.040462 | 0.01013 | 0.023572 | 5 |
| L44 | 0 | 0 | 0.004616 | 0.08611 | 0.092315 | 5 |
| L45 | 0 | 0 | 0.051149 | 0.149886 | 0.065001 | 5 |
| L46 | 0 | 0 | 0.004616 | 0.018951 | 0.010375 | 5 |
| L47 | 0 | 0 | 0.004616 | 0.011422 | 0.004495 | 5 |
| L48 | 0 | 0 | 0.418435 | 0.004328 | 0.004495 | 5 |
| L49 | 0 | 0 | 0.004616 | 0.009835 | 0.010269 | 5 |
| L5 | 0 | 0 | 0.010196 | 0.004328 | 0.014815 | 5 |
| L50 | 0 | 0 | 0.004616 | 0.02089 | 0.067955 | 5 |
| L51 | 0 | 0 | 0.012896 | 0.027138 | 0.093648 | 5 |
| L52 | 0 | 0 | 0.004616 | 0.026964 | 0.010063 | 5 |
| L53 | 0 | 0 | 0.016912 | 0.029818 | 0.004495 | 5 |
| L54 | 0 | 0 | 0.013279 | 0.066639 | 0.08592 | 5 |
| L55 | 0 | 0 | 0.004616 | 0.014453 | 0.010403 | 5 |
| L56 | 0 | 0 | 0.081908 | 0.109959 | 0.01449 | 5 |
| L57 | 0 | 0 | 0.208968 | 0.045293 | 0.004495 | 5 |
| L58 | 0 | 0 | 0.021149 | 0.426855 | 0.004495 | 5 |
| L59 | 0 | 0 | 0.004616 | 0.015937 | 0.010164 | 5 |
| L6 | 0 | 0 | 0.004616 | 0.030896 | 0.009835 | 5 |
| L60 | 0 | 0 | 0.009532 | 0.019318 | 0.004495 | 5 |
| L61 | 0 | 0 | 0.011811 | 0.054225 | 0.004495 | 5 |
| L62 | 0 | 0 | 0.004616 | 0.004328 | 0.146372 | 5 |
| L63 | 0 | 0 | 0.004616 | 0.017885 | 0.009835 | 5 |
| L64 | 0 | 0 | 0.035906 | 0.019862 | 0.004495 | 5 |
| L65 | 0 | 0 | 0.012198 | 0.105591 | 0.004495 | 5 |
| UW-515 | 0 | 0 | 0.004615 | 0.004327 | 0.190000 | 5 |
| L67 | 0 | 0 | 0.09439 | 0.015413 | 0.041739 | 5 |
| L68 | 0 | 0 | 0.012192 | 0.230458 | 0.025617 | 5 |
| L69 | 0 | 0 | 0.004616 | 0.010757 | 0.009835 | 5 |
| L7 | 0 | 0 | 0.310341 | 0.004328 | 0.059687 | 5 |
| UW-687 | 0 | 0 | 0.160353 | 0.004327 | 0.131815 | 5 |
| L71 | 0 | 0 | 0.0272 | 0.004328 | 0.004495 | 5 |
| L72 | 0 | 0 | 0.004616 | 0.013479 | 0.011171 | 5 |
| UW-500 | 0 | 0 | 0.080000 | 0.004327 | 0.280000 | 5 |
| L74 | 0 | 0 | 0.004616 | 0.010749 | 0.011689 | 5 |
| L75 | 0 | 0 | 0.004616 | 0.011422 | 0.028559 | 5 |
| L76 | 0 | 0 | 0.012991 | 0.088187 | 0.15747 | 5 |
| L77 | 0 | 0 | 0.055944 | 0.104043 | 0.051243 | 5 |
| L78 | 0 | 0 | 0.004616 | 0.030896 | 0.011239 | 5 |
| L79 | 0 | 0 | 0.004616 | 0.068988 | 0.011478 | 5 |
| L8 | 0 | 0 | 0.703137 | 0.182137 | 0.0268 | 5 |
| L80 | 0 | 0 | 0.004616 | 0.049003 | 0.010709 | 5 |
| L81 | 0 | 0 | 0.039794 | 0.034143 | 0.034799 | 5 |
| L82 | 0 | 0 | 0.034737 | 0.087857 | 0.015817 | 5 |
| L83 | 0 | 0 | 0.004616 | 0.030896 | 0.009835 | 5 |
| L84 | 0 | 0 | 0.017746 | 0.017306 | 0.012474 | 5 |
| L85 | 0 | 0 | 0.027353 | 0.332426 | 0.090025 | 5 |
| L86 | 0 | 0 | 0.004616 | 0.054444 | 0.009835 | 5 |
| L87 | 0 | 0 | 0.044169 | 0.011422 | 0.013049 | 5 |
| L88 | 0 | 0 | 0.011215 | 0.004328 | 0.004495 | 5 |
| UW-539 | 0 | 0 | 0.004615 | 0.160000 | 0.560000 | 5 |
| L9 | 0 | 0 | 0.229918 | 0.004328 | 0.004495 | 5 |
| UW-907 | 0 | 0 | 0.490000 | 0.730000 | 0.100000 | 5 |
| UW-732 | 0 | 0 | 0.050000 | 0.540000 | 0.190000 | 5 |
| UW-730 | 0 | 0 | 0.004616 | 1.790000 | 0.050000 | 5 |
| L93 | 0 | 0 | 0.004616 | 0.031672 | 0.010764 | 5 |
| L94 | 0 | 0 | 0.004616 | 0.037286 | 0.009956 | 5 |
| L95 | 0 | 0 | 0.004616 | 0.05437 | 0.011604 | 5 |
| UW-535 | 0 | 0 | 0.040231 | 0.004327 | 0.176418 | 5 |
| L97 | 0 | 0 | 0.010753 | 0.093957 | 0.061095 | 5 |
| L98 | 0 | 0 | 0.004616 | 0.030896 | 0.016752 | 5 |
| UW-509 | 0 | 0 | 0.004615 | 0.500000 | 0.220000 | 5 |

**Supplementary Table 4**. Linear regression analysis shows that LAM concentration positively correlates with (p-value 0.000) with urinary protein content. C.I. confidence interval.


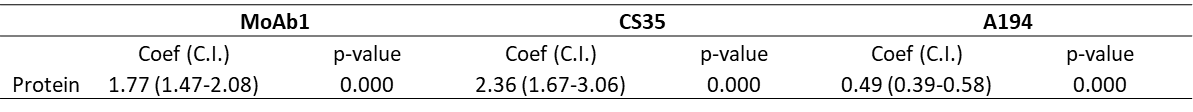

Supplement: Supplementary file 1 — Supplementary file1 [file 41598_2020_70669_MOESM1_ESM.docx]
